# Additive manufacturing of metals: a brief review of the characteristic microstructures and properties of steels, Ti-6Al-4V and high-entropy alloys

**DOI:** 10.1080/14686996.2017.1361305

**Published:** 2017-08-25

**Authors:** Stéphane Gorsse, Christopher Hutchinson, Mohamed Gouné, Rajarshi Banerjee

**Affiliations:** ^a^ CNRS, ICMCB, Univ. Bordeaux, Pessac, France; ^b^ ENSCBP, Bordeaux INP, Pessac, France; ^c^ Department of Materials Science and Engineering, Monash University, Clayton, Australia; ^d^ Woodside Innovation Centre, Monash University, Clayton, Australia; ^e^ Department of Materials Science and Engineering, University of North Texas, Denton, USA

**Keywords:** Additive manufacturing, 3D printing, microstructure, Ti alloys, steels, high entropy alloys, 10 Engineering and Structural materials, 106 Metallic materials

## Abstract

We present a brief review of the microstructures and mechanical properties of selected metallic alloys processed by additive manufacturing (AM). Three different alloys, covering a large range of technology readiness levels, are selected to illustrate particular microstructural features developed by AM and clarify the engineering paradigm relating process–microstructure–property. With Ti-6Al-4V the emphasis is placed on the formation of metallurgical defects and microstructures induced by AM and their role on mechanical properties. The effects of the large in-built dislocation density, surface roughness and build atmosphere on mechanical and damage properties are discussed using steels. The impact of rapid solidification inherent to AM on phase selection is highlighted for high-entropy alloys. Using property maps, published mechanical properties of additive manufactured alloys are graphically summarized and compared to conventionally processed counterparts.

## Graphical abstract

**Figure:**
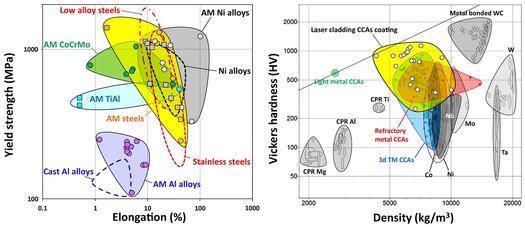

## 1. Introduction

Additive manufacturing (AM) is the process of fabricating objects layer by layer from 3D numerical models, as opposed to traditional subtractive manufacturing technologies. AM technologies for metals are numerous and include selective laser melting (SLM), selective electron beam melting (SEBM) and laser metal deposition (LMD), also called direct laser fabrication (DLF). They all have in common the local melting of a powder layer which is then rapidly solidified. In SLM [1], a laser beam scans and selectively melts a layer of powder. After exposure of the powder bed, another layer is applied and the process is repeated layer by layer until the completion of the part. Layer thicknesses range between 20 μm and 100 μm. Most laser-based systems have a maximum build rate of about 70 cm^3^/h and a build volume limited to 400 × 400 × 400 mm^3^. The SEBM process is similar to SLM with the difference that an electron beam is used instead of the laser to preheat and fuse the powder bed layers in a vacuum chamber [7,8]. SEBM has higher building rates (up to 100 cm^3^/h) but inferior surface finish (15–35 Ra instead of 4–11 Ra for SLM). LMD is an additive manufacturing process in which the part is cladded layer by layer [8]. Instead of melting selectively the material previously deposited on a powder bed, the powders are carried by an inert gas into a laser beam where they melt, and are fed onto the workpiece where they fuse with a thin surface layer previously deposited. This technique presents the advantages of having no restriction on the build size, the highest build rates (up to 300 cm^3^/h) of the AM techniques, and allows the fabrication of graded and hybrid materials by simultaneously feeding two different filler materials. Surface roughness ranges between 10 Ra and 200 Ra.

As a tool-free, cost-efficient and digital approach to manufacturing, AM of metals offers many key benefits that could change the industrial paradigm in various sectors such as aerospace, automotive, energy, medical, tooling and consumer goods: [Fig F0001]

**Figure 1. F0001:**
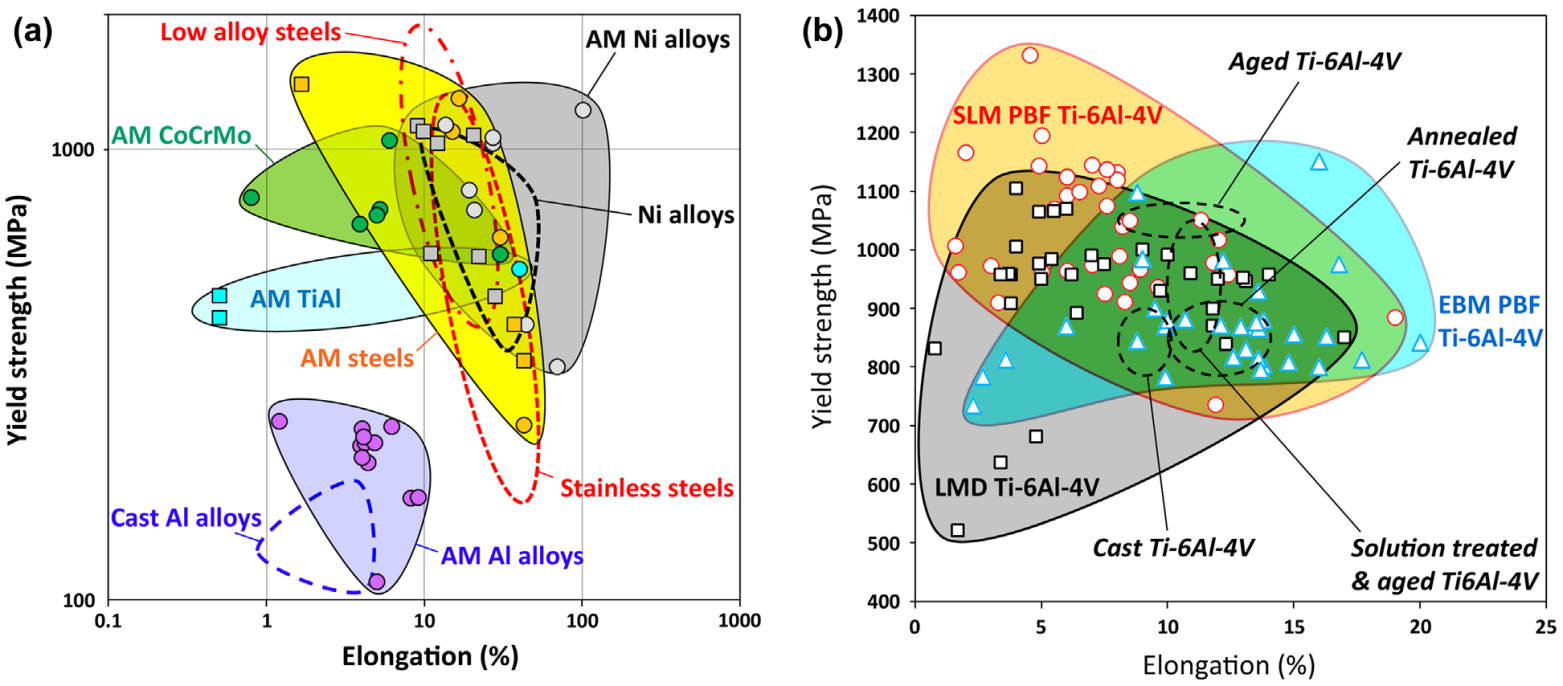
Materials property space for room temperature yield strength vs elongation of additively manufactured alloys [8] and conventionally manufactured alloys (dashed lines), including (a) steels, Ni alloys, Al alloys, TiAl and CoCrMo, and in (b) Ti-6Al-4V alloys (PBF stands for powder bed fusion).

- provides characteristics superior to those of cast parts and approaches those of forged parts (Figure 1),
- affords the creation of complex 3D geometries, such as architectured lattice structures, topologically optimized structures, recesses for configurational cooling channels, etc., that are not possible to achieve with other traditional processes,
- net shape process which dramatically shortens the fabrication time and cost due to the elimination of production and assembly steps, and reduces the material waste and environmental impact,
- enables low-volume production and mass personalization,
- permits 3D functionalization and surface engineering.

Metals which can be processed by AM must meet two main criteria: good weldability to avoid cracks during solidification, and raw materials available as spherical powders with a size of a few tens of microns in order to achieve good packing density and homogeneity of the powder deposition. Fewer than 50 different alloy compositions are currently available as atomized powders from about 15 suppliers, and are used in AM at various technology readiness levels (TRL). In terms of volume production and level of adoption, the most common and mature metallic alloys processed by AM are, in descending order, from materials being used to fabricate components for commercial usage to alloys subject to research [9]:

- Ni-based superalloys (Inconel 625, Inconel 718 and Hastelloy X) with TRL 7–9,
- Co-Cr alloys (Co28Cr6Mo) with TRL 7–9,
- tool steels (H13 and Maraging 300) with TRL 9,
- stainless steels (316L and 17–4PH) with TRL 7–8,
- Ti-based alloys (commercial purity grade 1 and grade 2, Ti-6Al-4V, Ti-6Al-4V ELI) with TRL 7–9,
- Al-based alloys (AlSi12, AlSi10Mg, AlSi7Mg0.6, AlSi9Cu3, AlSi5Cu3Mg, 1050A, 2017A, 2219, 6061, 7020, 7050, 7075 and 5083) with TRL 4–8,
- precious metals (Au, Ag),
- refractory metals (W, Ta),
- Cu-based alloys, intermetallic (titanium aluminide), and low alloy steels (AISI 4140) with TRL 4–5.

This choice of materials for AM is rather limited, and there is strong need both for (1) increasing the maturity of available materials to enable the fabrication of critical parts or the production in large volumes, and (2) expanding the variety of compatible materials, in order to fill the gap that remains between what can be done in AM in comparison with conventional casting and forging processes.

Because of the rapid cooling rates and directional solidification, metals produced by additive manufacturing have structures, microstructures, and three-dimensional multiscale architectures that differ from their cast and wrought counterparts. Fine grains, anisotropic microstructures with elongated grains, non-equilibrium microstructures (metastable phases, solute trapping, non-equilibrium compositions) and metallurgical defects, such as porosity due to unmelted powders and gas entrapment, are among the distinguishing bulk microstructural features of metals processed by AM [10]. The surfaces of metallic components fabricated by AM also exhibit characteristic features which can include weld tracks, protruding unmelted powder particles or ejected molten droplets and recesses. Due to the different bulk and surface structures in AM metals, the mechanical behavior may differ substantially from conventionally processed materials [11] as illustrated for various types of alloys in Figure 1. It is thus of prime necessity to better understand the complex relationships between powders’ metallurgical parameters, processing, microstructure and mechanical properties.

In this paper, we will review the microstructures of three selected alloy classes produced using AM to clarify the engineering paradigm relating process–microstructure–property. These three alloy classes are chosen to span the maturity (or TRL) of materials currently being produced by AM and to highlight particular microstructural features resulting from AM.

Ti-6Al-4V is first discussed because it has been very heavily studied in the context of AM due to its cost advantages compared to conventional machining. The typical microstructural defects such as porosity, lack of fusion, hot-tearing, etc., and the elongated grain shapes resulting from SLM are discussed in this section using Ti-6Al-4V as an example. The elongated grain shapes often observed in SLM are due to the very large temperature gradient and rapid solidification, and this process also results in a high as-built dislocation density. The important effects of this in-built dislocation density are discussed in Section 2 using SLM of steels as an example. Whereas the porosity resulting from AM of metals is rightly highlighted as a key factor in the damage properties obtained such as fatigue, in Section 2 we also highlight the important role of the surface roughness on the fatigue response of steels fabricated by SLM.

The rapid solidification inherent in the SLM process leads to highly non-equilibrium microstructures and can provide an interesting tool to aid phase selection during alloy fabrication. In Section 3, the stability of phases formed in steels by AM is discussed, and this concept is then extended significantly in Section 4 which focuses on high-entropy alloys. High-entropy alloys (HEAs) have only recently been fabricated using AM, for both bulk materials and as claddings on other materials, but they are particularly well suited to AM, and we may expect this field to grow significantly in the coming years. The properties that are obtained and the effects of the processing on the microstructures of HEAs are discussed.

## 2. Microstructure and mechanical properties of Ti-6Al-4V produced by selective laser melting

In this section, we will focus on Ti-6Al-4V alloys due to their broad applications in biomedical devices, aerospace, marine and offshore applications and their fracture resistance, fatigue behavior, corrosion resistance and biocompatibility [12,13]. Emphasis is placed on the effect of metallurgical defects on the mechanical properties of Ti-6Al-4V.

### 2.1. Metallurgical defects

**Figure 2.**
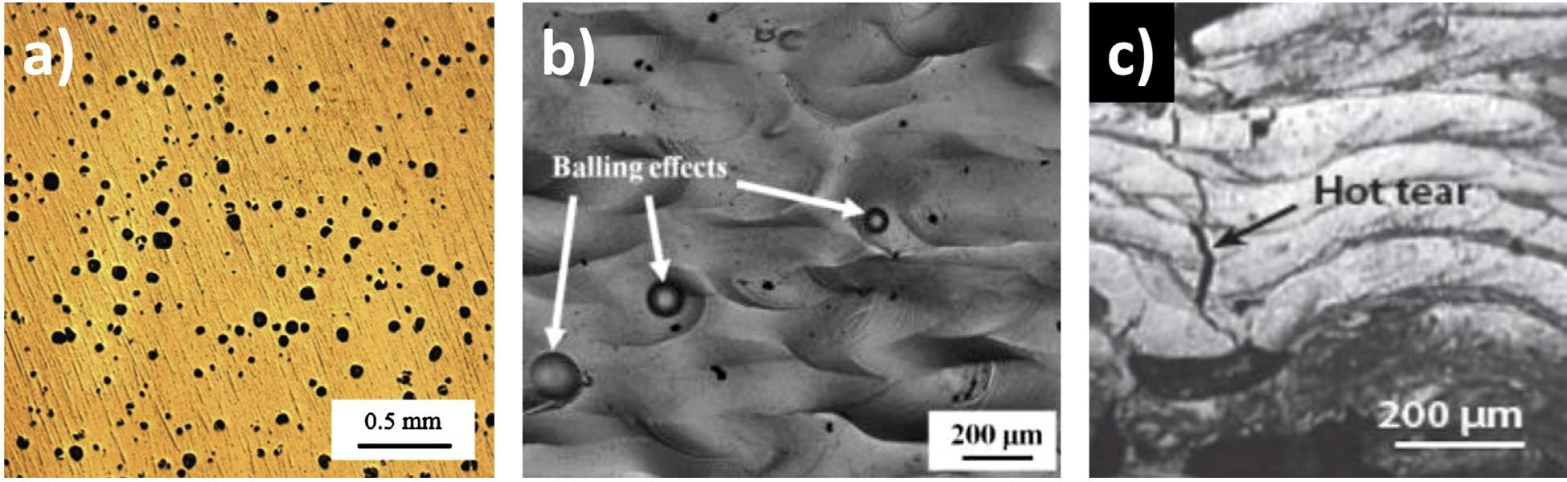
Examples of defects that can form in SLM materials: (a) porosity formed in SLM Ti-6Al-4V [14], (b) balling [16] and (c) hot tears [17].



Materials produced by SLM contain defects that affect their mechanical properties [10]. Several sources can be identified: (1) unmelted or partially melted particles of powders, (2) lack of fusion, (3) delamination between adjacent passes or previous deposited layers, and (4) entrapment of gases during manufacturing [10,14–17]. Figure 2 gives some examples of defects that can form in SLM materials. Many researchers have studied the effect of process parameters on defect generation in SLM [10,14,18,19]. A link between the energy density and the volume fraction of porosity was shown. The energy density represents the applied energy per unit volume during a powder bed fusion process and can be expressed as:

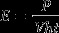



where P is laser power, V is scan speed, h is hatch spacing and t is layer thickness.

**Figure 3.**
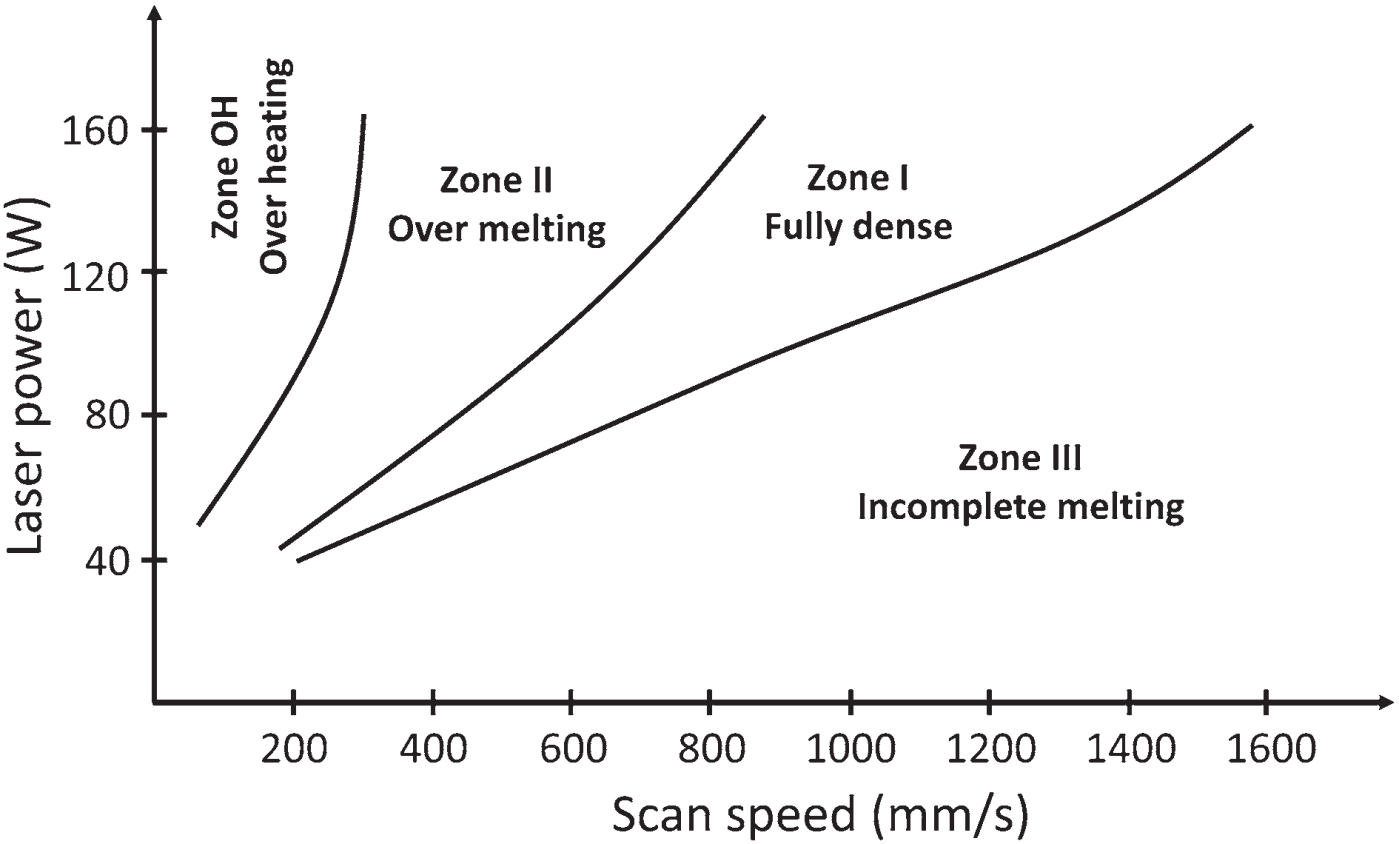
Process window for SLM-produced Ti-6Al-4V samples [14].

Porosity can form due to insufficient energy input or due to the use of excessive energy [20]. For a given layer thickness and hatch spacing, the process window can be divided into four melting zones [14] (Figure 3).

The sample is relatively free from porosity in zone I named ‘fully dense zone’. The sample contains measurable porosity in zones II and III named, respectively, ‘over melting zone’ and ‘incomplete melting zone’. The porosities induced by process parameters of zone II are caused by the use of excess energy, while those of zone III are induced by insufficient energy input. Finally, a zone termed ‘over heating zone’ is obtained for very low scan speed and high laser power. The excess energy is such that it is difficult to elaborate any samples in these process conditions. The shape of the defects is also an indicator. Indeed, the near-spherical defects are usually associated either to gas bubbles when a high energy laser is applied or to a destabilization of the liquid interface. In the first case, the vaporization of low-melting-point constituents within the alloy occurs. In the second case, it can lead to a balling effect as shown in Figure 2(b). The above discussion excludes lack of fusion and layer delamination. Regarding lack of fusion, it can be generated at both low and high energy densities. At low energy density, it is attributed to the size of the molten pool which can be smaller than the adjacent passes of the laser path. At high energy density, thermal distortions are identified as a probable source. In addition, the formation of hot tears is a consequence of the material response to stress at elevated temperature (Figure 2(c)).

Besides the process parameters, the metallurgical parameters of powders such as composition, size distribution, morphology and surface characteristics play a role in defect formation [21]. One of the requirements to successively deposit uniform layers is to enhance the powder’s flowability [22]. Both the powder shape and size distribution control this property [23]. Furthermore, the energy required to melt all incoming powder is expected to depend on the size of particles. Larger particles require higher laser power for melting due to lower heat transfer.

### 2.2. Macrostructure and microstructure

Selective laser melting can be seen as a very rapid solidification process. The high temperature gradients generated lead to complex macrostructure and microstructure. The manufacturing of Ti-6Al-4V components by SLM involves the melting of a small volume of powders. The liquidus temperature of Ti-6Al-4V is around 1650 °C, and its solidus temperature is around 1605 °C [24]. The liquid metal pool temperature depends on the laser power and process conditions. For a power laser of 350 W, a deposition rate of 0.13 g/s and a layer thickness of 508 μm, the metal pool temperature ranges from 2000 °C to 2500 °C [25]. The cooling rate in the melt pool of Ti-6Al-4V, which is one of the key parameters in order to control subsequent phase transformations to room temperature, is between 12,000 °C/s and 40,000 °C/s depending on the applied energy [26].

**Figure 4.**
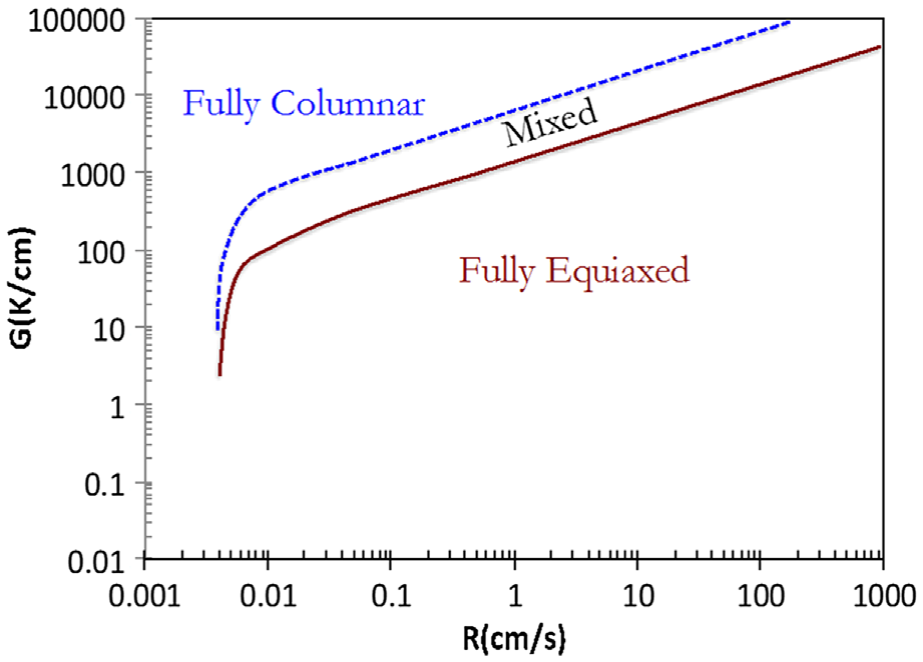
The Ti-6Al-4V solidification map showing the predicted values of the nature of solidification macrostructure (fully columnar, mixed and fully equiaxed) as a function of both the thermal gradient G and the solidification rate velocity R [29].

Ti-6Al-4V components produced by SLM solidify first as *bcc*-*β* phase. The solidification macrostructure is composed of both columnar and equiaxed grains whose architecture can be complex [10,27,28]. The conditions for the formation of fully columnar and fully equiaxed structures can be represented in terms of a solidification map as shown in Figure 4 [29]. A fully columnar structure is observed for low values of solidification rate velocity (R) and high value of thermal gradient (G), and a fully equiaxed structure is observed in the opposite case.

**Figure 5.**
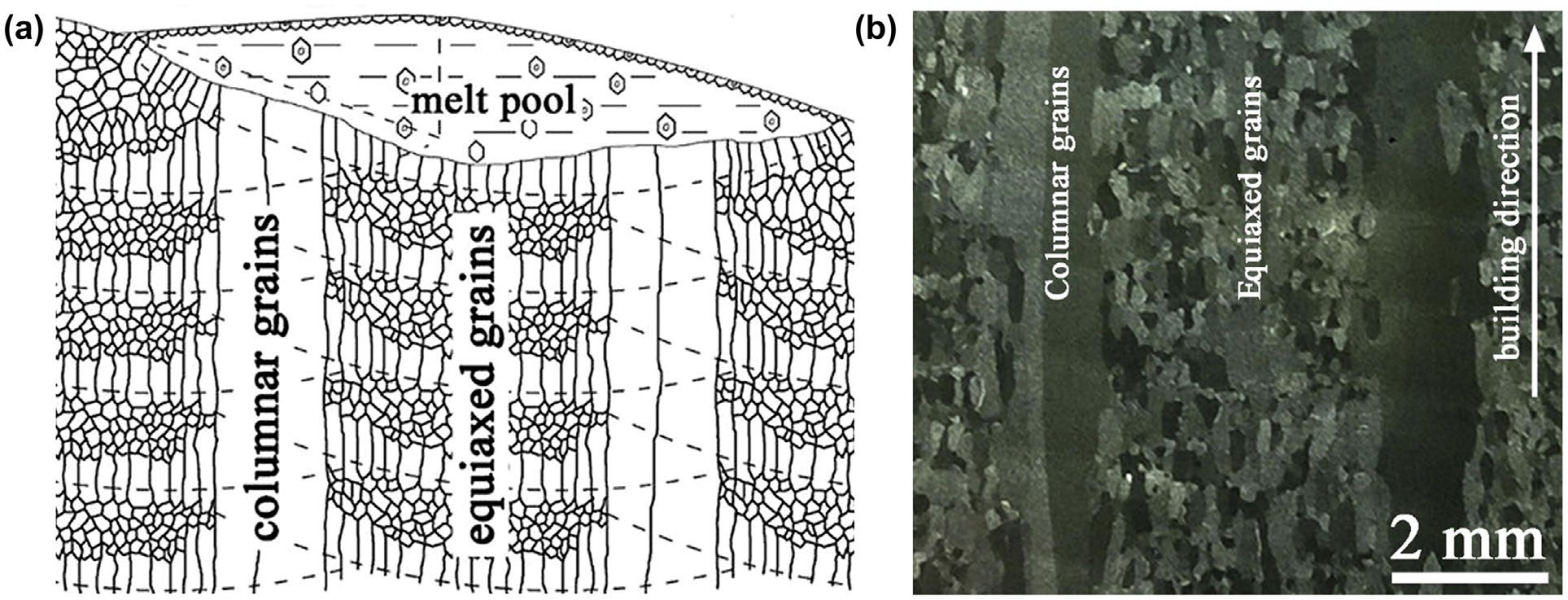
Formation of the ‘steel-bar reinforced concrete-like’ mixed grain structure composed by coarse continuous columnar grains and fine equiaxed grain during the layer-by-layer additive manufacturing. (a) Schematic illustration, (b) optical micrograph (from [28]).

In titanium components produced by laser melting deposition, the as-solidified morphology results from the competition between the nucleation of new equiaxed grains on partially melted powder particles and the pool-bottom epitaxial growth of large columnar grains [28]. All being the same, mass deposition rate is known to be a critical processing parameter. In some specific cases, a ‘steel-bar reinforced concrete-like’ mixed grain structure can even be obtained (Figure 5).

The <100> fiber solidification growth direction was reported to tend to be aligned with the direction of the maximum thermal gradient that corresponds to the SLM built direction [30]. In as-built SLM specimens, columnar grains correspond to cubic *β* grains. Upon cooling, *β* phase transforms to α’-martensite for high cooling rate (above 410 °C/s), and to α for slow cooling rate (below 20 °C/s) [31,32]. In both cases, the newly formed hexagonal phase has a clear crystallographic relationship with parent *β* phase.



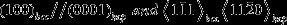



**Figure 6.**
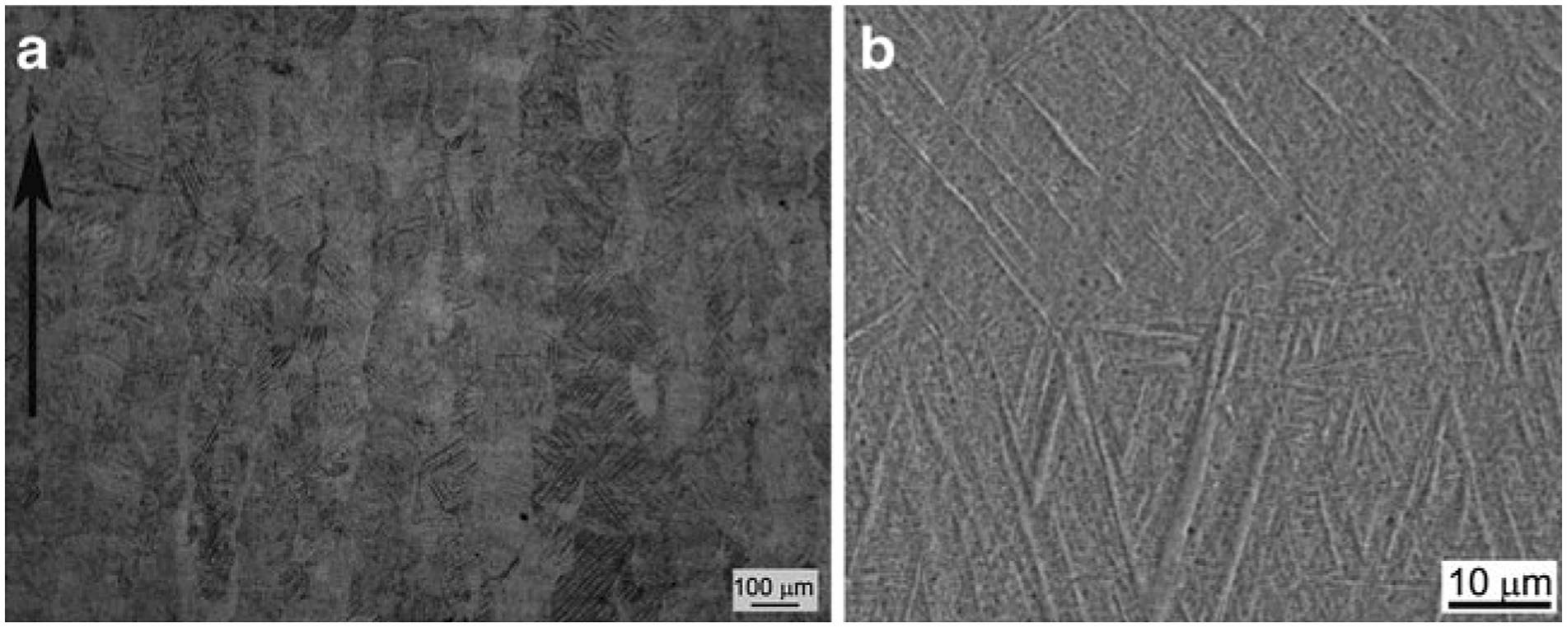
(a) Optical micrograph of Ti-6Al-4V part built in a vertical direction. (b) High-magnification scanning electron microscopy (SEM) image showing α’-martensite needles formed from the columnar *β* grains [33].

Typically, because of the high cooling rate during the SLM process, the α’ martensitic transformation occurs [33] (Figure 6). The orientation relationship between α’ and *β* dictates the α’ preferential growth orientation, and martensitic needles (or laths) are generally observed to be inclined about 40° with respect to the build direction [33,34]. Furthermore, a high dislocation density and stacking faults were observed by transmission electron microscopy (TEM) and electron diffraction [35,36]. The observed tensile 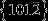 twinning was suggested as a possible mechanism for accommodation of thermal stresses during manufacturing [35]. Twinning is not usually considered as the dominating deformation mechanisms in conventional Ti-6Al-4V [37].

### 2.3. Mechanical behavior

The presence of a fine and fully α’-martensite structure in as-built SLM samples of Ti-6Al-4V is a consequence of rapid cooling and results in high tensile strength (>1000 MPa), high yield strength (>900 MPa) and a low ductility (total elongation < 8%) [8,38,39]. The improvement of mechanical properties and especially the total tensile elongation to failure requires the transformation of the α’-martensite into equilibrium (α+*β*) phase. This generally requires some specific post-SLM heat treatments. However, it was recently shown that *in situ* α’-martensite decomposition can be realized during SLM to produce an ultrafine (α+*β*) lamellar structure [40]. The total elongation to failure is about 11.4% while maintaining high yield strength above 1100 MPa, superior to both conventional SLM-fabricated Ti-6Al-4V and conventional mill-annealed Ti-6Al-4V containing globular (α+*β*) structure. It is worth noting that the formation of the acicular α’-martensite or ultrafine lamellar (α+*β*) was found to depend on the energy density E; reducing E from 50.6 to 33.7 J/mm^3^ leads to a change from ultrafine lamellar (α+*β*) to acicular α’-martensite. As a consequence, a decrease of E favors the formation of α’-martensite structure. This clearly shows a promising pathway to develop high-strength and ductile titanium alloys.

**Figure 7.**
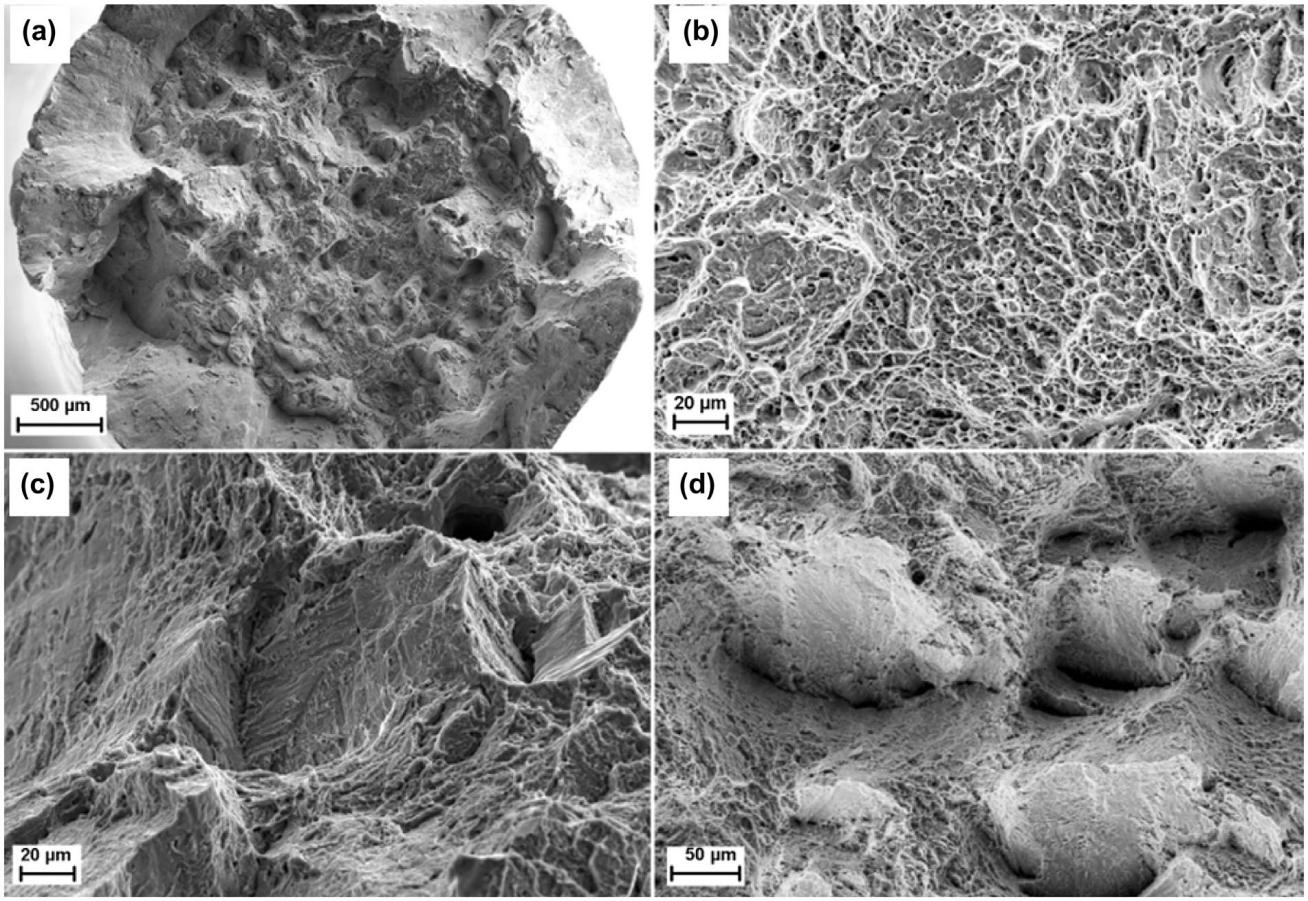
Fracture surfaces after tensile tests of as-built SLM Ti-6Al-4V specimens: (a) cup-and-cone, (b) dimples, (c) and (d) quasi-cleavage facets (from [35]).

For both horizontal and vertically built Ti-6Al-4V samples, tensile failure is a mix of ductile and brittle modes [35,38]. The fracture surfaces after tensile tests of as-built specimens (Figure 7) show a cup-and-cone shape of the necking region and a dimple rupture associated with pore coalescence and a ductile mode (Figure 7(a) and 7(b)). In some cases some quasi-cleavage facets more representative of both brittle and ductile modes can be observed. It is worth noting that martensite needles (or laths) are often visible on the quasi-cleavage surfaces and the cracks are often deviated by the prior *β* grain boundary [35]. It is thus suggested that boundaries act as crack deflectors and prevent catastrophic failure. The fatigue trials lead to the same conclusion. Indeed, in that case it is also supposed that the high density of boundaries acts as obstacles for propagation of small cracks [24,41,42]. Surprisingly, the crack propagation rate was measured to be faster for cracks larger than 1 mm without any clear explanation [41,42]. The macro fracture surface is flat without any obvious necking. However, the micro fracture surfaces are characterized by microvoid coalescence resulting in a dimpled appearance, cleavage facets and lots of opened-up pores. These indicate clearly a mixed mode of ductile and brittle fracture [43,44]. It is observed that cracks initiate from internal pores in the subsurface and propagate radially outwards from the defect [38,44]. The crack initiation at the subsurface was attributed to the presence of a stress-raising defect. The fatigue properties are thus very sensitive to the presence of porosity. Indeed, lower uniaxial fatigue performance has been reported in Ti-6Al-4V SLM samples due to porosity [45,46]. More particularly, the fatigue strength seems to be more affected by micron-sized pores [47]. The fatigue performance depends also on surface conditions and microstructural state. Most of the fatigue data reported on Ti-6Al-4V additively manufactured in both as-built and heat-treated conditions have been assessed in two recent reviews [48,49]. The analysis of the data shows that the surface roughness and residual stresses can severely degrade the high-cycle fatigue performance by providing fatigue initiation sites. It is worth noting that unmelted particles at the surface reduce the fatigue lifetime by an order of magnitude in comparison to subsurface crack initiation from unmelted particles in the bulk. As a consequence, post surface treatments are essential to achieve superior fatigue performance. Furthermore, anisotropic fatigue performance can be observed in additively manufactured Ti-6Al-4V often attributed to the columnar prior *β* grain structures, non-uniform distribution of pores and other microstructural features. Finally, it can be noted that the fatigue strength of additively manufactured Ti-6Al-4V scatter broadly and can reach levels comparable to, or even better than, those of the mill-annealed Ti-6Al-4V.

**Figure 8.**
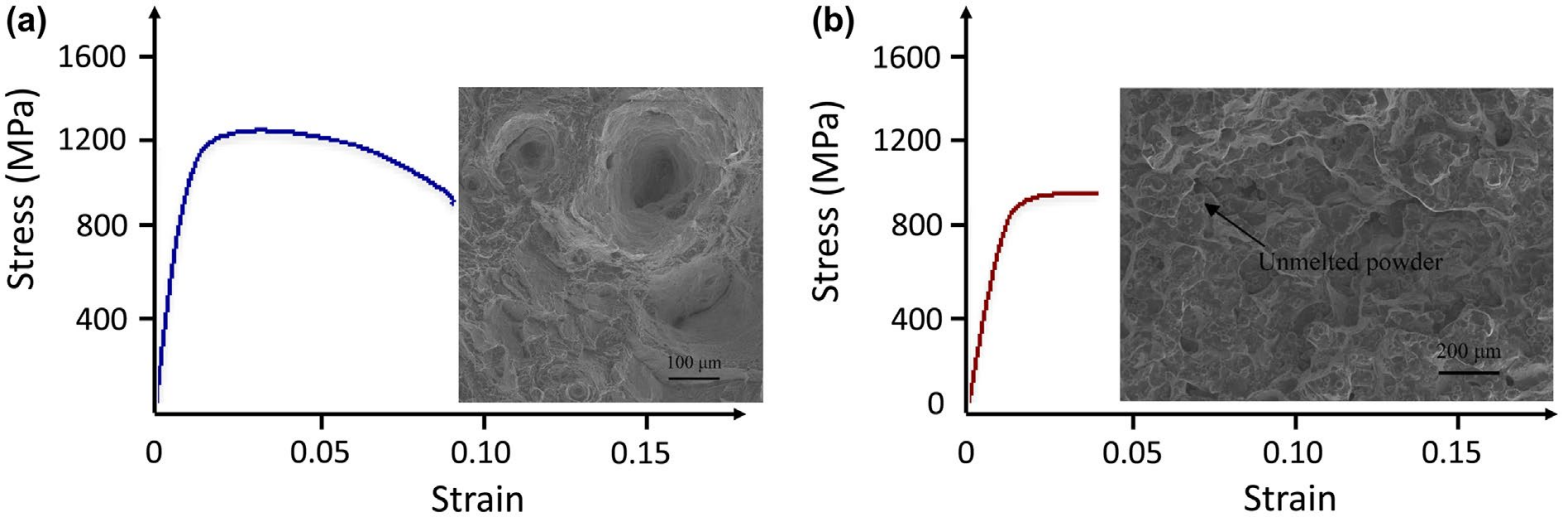
Tensile curves and fracture surfaces of SLM Ti-6Al-4V specimens: (a) with very low volume fraction of porosity (0%), (b) with volume fraction of porosity of about 5% (from [20]).

The role of metallurgical defects in the mechanical properties such as hardness and the stress/strain response has also been highlighted. The simplest mechanical property to be analyzed is hardness. It was shown that both the volume fraction of porosity and its size influences hardness. A significant decrease of hardness was recently observed for high-volume fractions of porosity (5%) and larger pores [20]. It was shown that the type of defects plays a key role on the tensile properties. Defects caused by insufficient energy input have a strong influence on the tensile curve from 1% of porosity [20]. When the porosity level is around 5%, the mechanical properties including ultimate tensile strength, elongation and Young’s modulus are all strongly deteriorated (Figure 8). The fracture surface of samples with high levels of porosity (Figure 8(b)) exhibits numerous unmelted powder particles, while few discernible voids were observed for very low levels of porosity (Figure 8(a)).

The effects of build direction and orientation on mechanical properties remain unanswered. Some works report that the orientation has no clear effect on ultimate tensile strength or yield stress but influences the elongation [50]. The vertically built samples were tested with the columnar grains parallel to the stress axis, and the horizontally built samples were tested with the stress axis perpendicular to the length of the columnar grains. The difference in the elongation was thus mainly attributed to the fact that the columnar grains are along the length of the tensile samples of the vertically built samples. This is consistent with the scanning electron microscopy (SEM) observation on fracture surfaces. Indeed, the horizontally built samples exhibit more planar and faceted fracture morphology than the vertically built samples. However, other works report that ultimate tensile strength (UTS) for the horizontally built samples are higher than the UTS for the vertically built samples [33]. This anisotropy of tensile properties was attributed to the orientation of the defect with respect to the loading direction [51,52]. If the defects are perpendicular to the loading direction, the defects are expected to open at relatively low stress levels. If the defects are parallel to the tensile loading axis, the opening of these defects becomes more difficult. It is worth noting that size, morphology and nature of porosities also influence the mechanical properties [20]. For instance, defects caused by insufficient energy input have a strong influence on mechanical properties even when present in low amounts. In contrast, defects caused by excessive energy input are less detrimental to mechanical properties.

## 3. Microstructure development in selective laser-melted steels

As highlighted in Section 2 on Ti-6Al-4V, one of the first order microstructural parameters that is of concern during any additive manufacturing process is porosity. Even small fractions of porosity (<1%) will affect damage properties such as fatigue, and larger porosities (>5%) can detrimentally affect the monotonic mechanical response significantly. However, there is a great deal of work currently underway on the identification of appropriate printing conditions for the different additive manufacturing approaches, and, for many alloy systems, printing conditions that result in porosities less than 1% have been identified. These processing considerations for consolidation of dense products have previously been reviewed in detail (e.g. [8]) and were discussed in Section 2 in the context of Ti-6Al-4V. In this section emphasis will instead be placed on the microstructural features generated in almost fully dense additively manufactured steels. Selective laser melting will be the process discussed, and stainless steels and maraging steels will be used as examples to highlight several important features of the microstructures of additively manufactured steels that have received much less attention than the porosity and which deserve further focus as the field moves forward.

### 3.1. Grain and dislocation structures in SLM stainless steels

**Figure 9.**
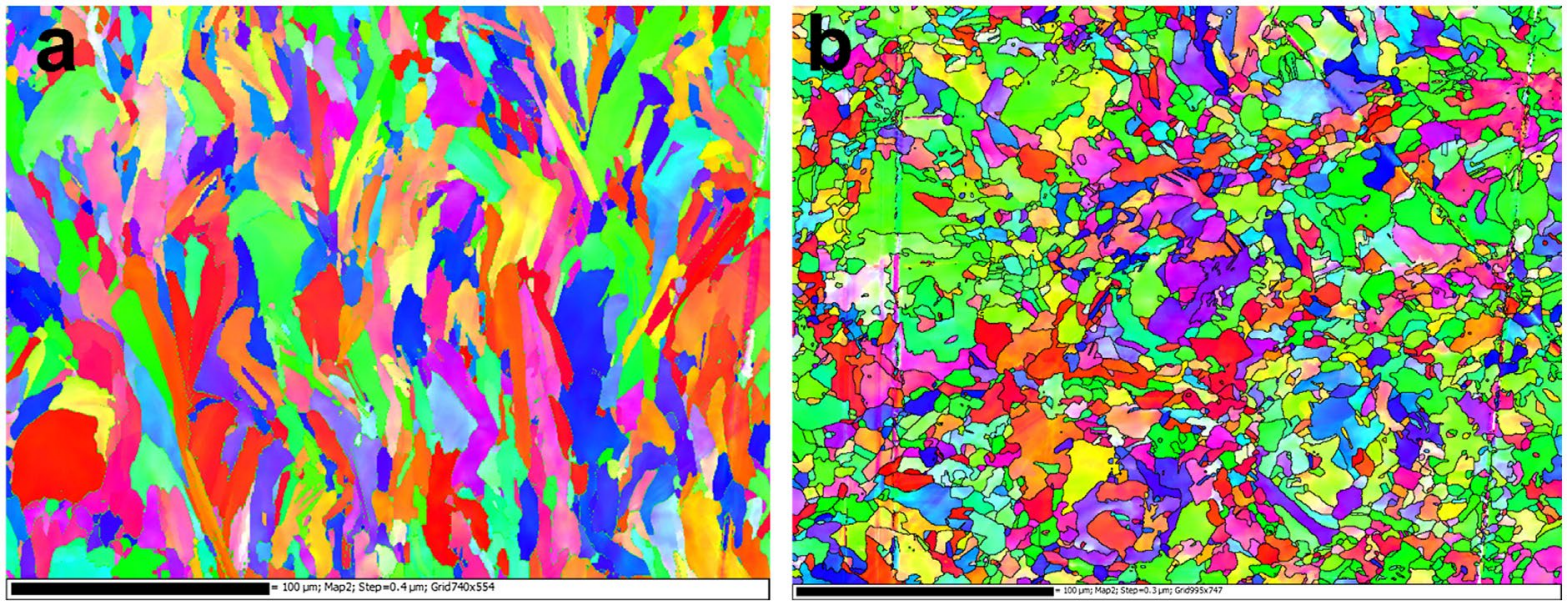
EBSD maps (inverse pole figure: blue is 111, green is 101 and red is 001) taken from as-built 316L prepared using SLM on an EOS280 using factory-recommended print settings: (a) the build direction is vertical, and (b) the plane of the image is perpendicular to the build direction.

The defining feature of the SLM process is the rapid solidification of a small melt pool under conditions of anisotropic heat removal. This gives rise to the types of grain microstructures shown in Figure 9. This electron backscattered diffraction (EBSD) map is from a SLM 316L stainless steel built on an EOS 280 machine (Electro-Optical Systems, Krailling, Germany). In Figure 9(a) the build direction is vertical, and Figure 9(b) is taken from a plane perpendicular to the build direction. The microstructure is 100% austenitic. In this case, the microstructure is almost fully dense, and the grains are elongated in the build direction due to the heat removal through the build plate during solidification. The width of the elongated grains (which is the finest dimension) is approximately 10 μm (Figure 9(b)), and this is significantly finer than the grain size typically observed in wrought 316L or the cast equivalent (CF3 M) (~30–60 μm). However, even this finest dimension of the elongated grains generated during SLM is not what we would refer to as ultra-fine-grained. This refinement in grain size will influence the mechanical response (and anisotropy), but if one considers the expected increase in Hall–Petch strengthening in going from a 50 μm to a 10 μm grain size for such a material, it is unlikely to explain the doubling of the yield strength that has been reported experimentally for SLM 316L (0.2% proof stress of ~600 MPa) [7,8] compared with its wrought (annealed) or cast equivalent (0.2% proof stress of 200–300 MPa).

**Figure 10.**
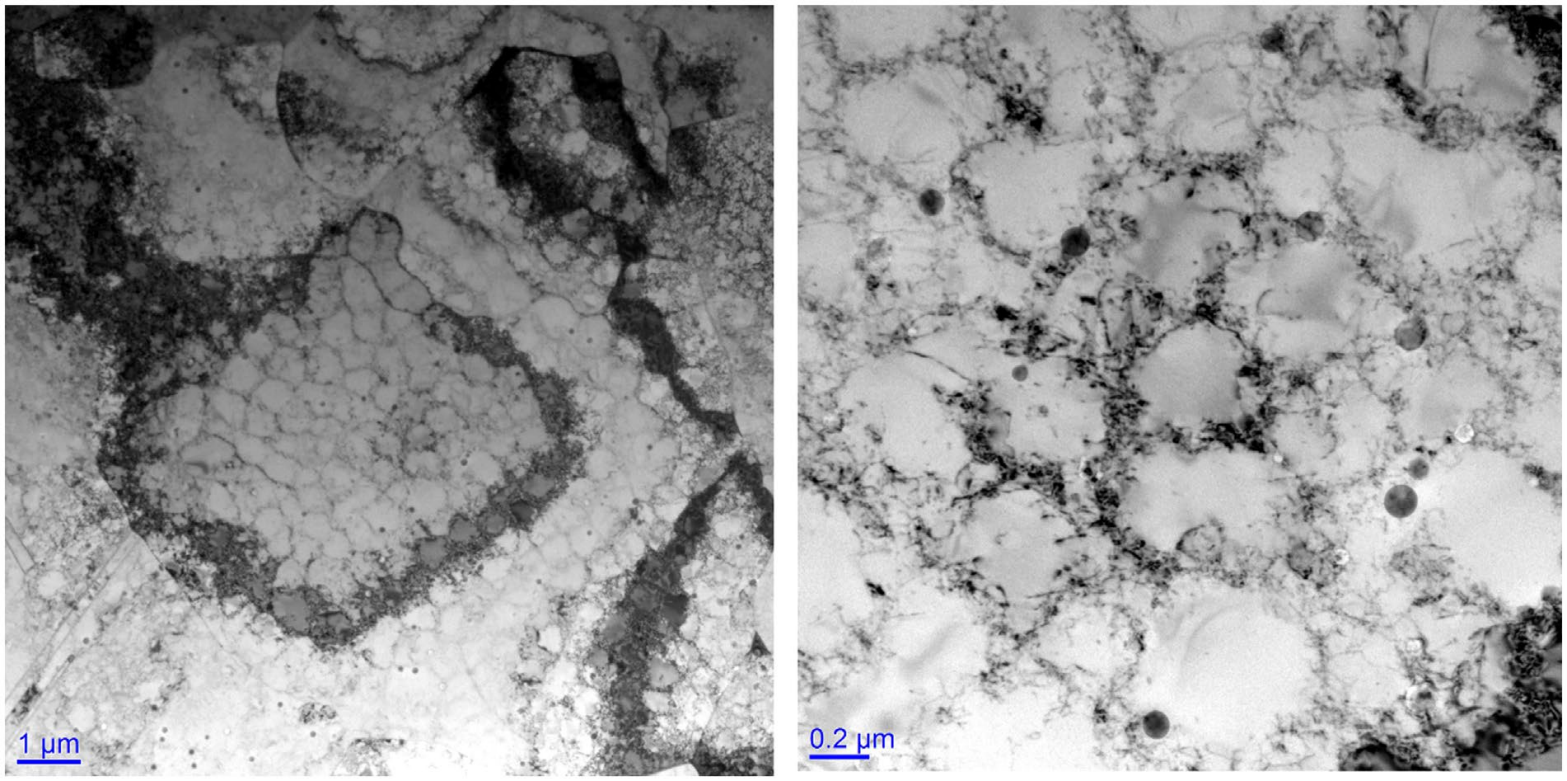
Transmission electron micrographs of dislocation substructures generated in as-built 316L prepared using SLM on an EOS280 using factory-recommended print settings.

If we examine in TEM the microstructure of an as-built SLM steel such as 316L, things are much more interesting. Examples of bright-field TEM micrographs are shown in Figure 10. These images are prepared from TEM foils sectioned normal to the build direction (i.e. the plane of the image is the same as that shown in Figure 10(b)). The characteristic feature of the as-built microstructure is a large dislocation density arranged as fine dislocation cells of diameter 200–400 nm. This is a very fine dislocation cell structure formed in an as-built SLM sample with similarities to the deformation substructures obtained after severe plastic deformation (SPD).

If the cell size is taken as 400 nm, one may make a rough estimate of a dislocation density of the order of 4×10^14^ m/m^3^. If one assumes a cell size of 200 nm, then an overall dislocation density of close to ~10^15^ m/m^3^ is obtained. These are very high dislocation densities obtained in as-built structures which correspond to the dislocation densities that would be obtained in heavily deformed metals. In comparison, the dislocation density of annealed wrought 316L, or the cast alloy CF3 M, will be of the order of 10^9^–10^10^ m/m^3^.

Two questions immediately come to mind: How can such a high dislocation density be obtained in as-built structures? And what is the effect of this high density on the mechanical response?

The qualitative explanation usually offered for the high dislocation density is that it is due to thermal contraction stresses during the rapid solidification, although it does not appear that a quantitative rationalization of this explanation has been demonstrated. One may test this by considering the thermal contraction strain during solidification. For most steels, solidification will occur around 2000 K, and we may estimate the linear thermal strain (Δε) of the solid as it cools to room temperature as:



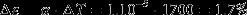



An upper estimate, assuming no recovery or other mechanisms of stress relief, for the dislocation density may be obtained by considering that all of this linear thermal strain is accommodated by the solid punching out dislocations as it cools.

If this thermal strain is fully accommodated by dislocations, then the upper estimate of the dislocation density can be estimated as:



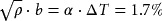



This leads to an upper estimate of the dislocation density of 5×10^15^ m/m^3^. This compares reasonably well with the estimates based on the cell size shown in Figure 10 and demonstrates quantitatively that such a high dislocation density is possible from thermal contraction alone. Although the cooling rate is very fast, it is not instantaneous, and some dislocation recovery and rearrangement will occur (at least enough for the dislocations to arrange into cells), and as a result the experimentally observed dislocation density is smaller than that calculated above.

There are two important consequences of this high dislocation density obtained in as-built SLM steels such as 316L. The first is a significant contribution of the forest dislocation density to the flow stress. This may be estimated using Taylor’s equation 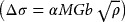 to be 110 MPa for a dislocation density of 4×10^14^ m/m^3^ and 175 MPa for a dislocation density of 1×10^15^ m/m^3^. For these calculations, a value of αM of 0.3 has been used for the *fcc* austenite.

**Figure 11.**
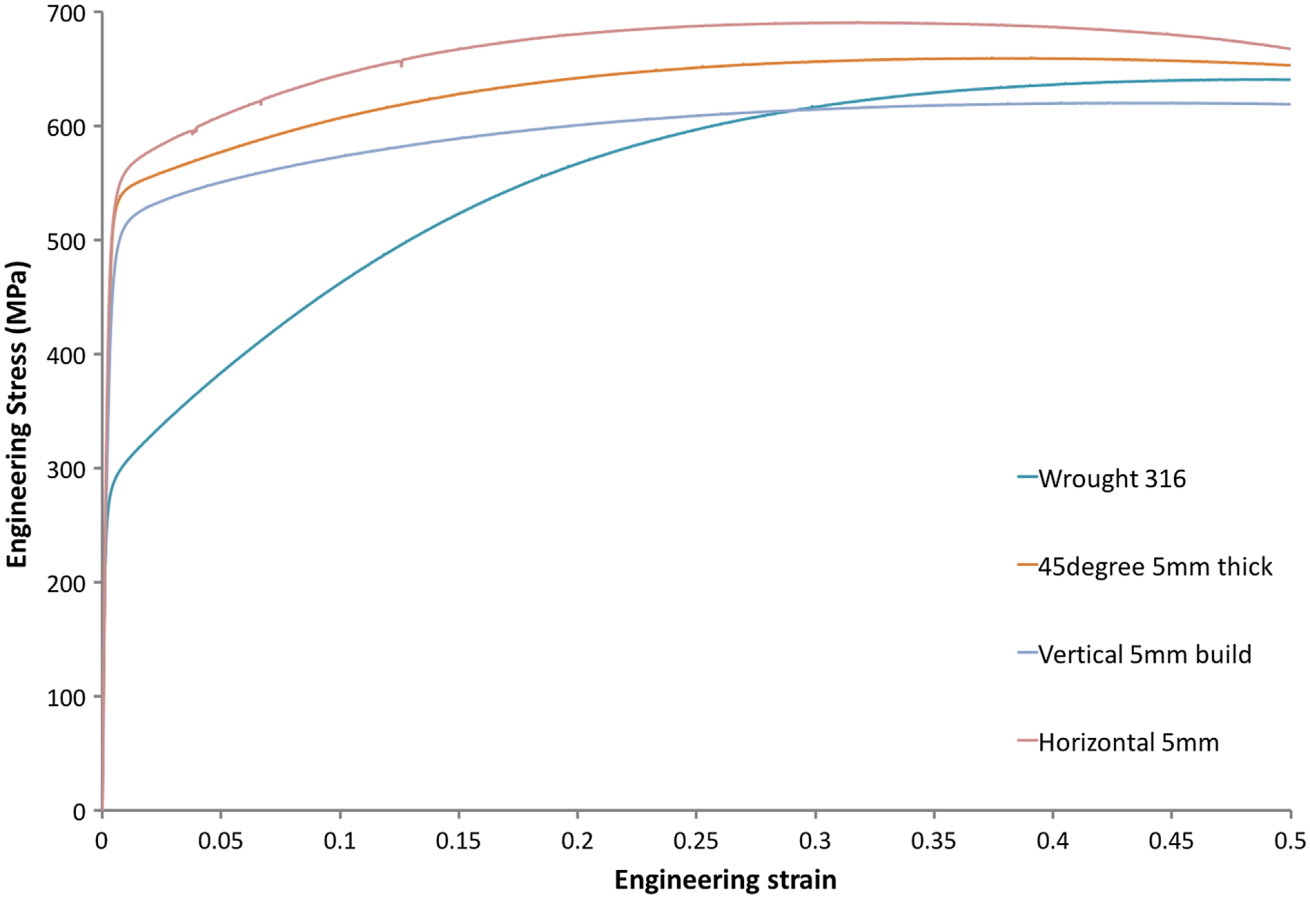
Engineering stress/strain curves for wrought 316L compared with as-built 316L fabricated on an EOS280 using factory default settings. The tensile sample was built directly and tested without any machining or surface treatments. The gauge length was 20 mm, and width and thickness 5 mm each.

Consider the stress/strain curves shown in Figure 11 for the as-built SLM 316L shown in Figures 9 and 10, compared with typical annealed wrought 316L. The 0.2% proof stress of the as-built vertically oriented 316L tensile sample is ~510 MPa compared with 290 MPa for the wrought 316L. The cast equivalent of wrought 316L (CF3 M) has basically the same monotonic mechanical properties as annealed wrought 316L. The difference is 220 MPa, of which approximately (110 to 175 MPa)/220 MPa = 50%–80% is likely due to the increased dislocation density present in the SLM 316L. It is true that the refined grain size generated in SLM metals will contribute to the increased strength of these products compared to wrought equivalents, but the more important contribution is from the high dislocation densities present in the as-built structures after SLM.

The effect of the grain refinement during SLM may be inferred from the orientation dependence of the stress/strain curves shown in Figure 11. Stress/strain curves are shown for the vertically oriented tensile sample builds, as well as samples built horizontally and at 45% to the build plate. Anisotropy is observed, and the effects are as expected – the horizontally oriented tensile sample has the largest yield strength because the finest dimension of the elongated grain structure will be oriented normal to the tensile axis. However, the difference in yield strength between the vertically built and horizontally built 316L is only ~50 MPa, which is a small fraction of the typical difference between the strengths of wrought or SLM 316L. Again, this highlights the key role of the dislocation density in the as-built SLM structures.

**Figure 12.**
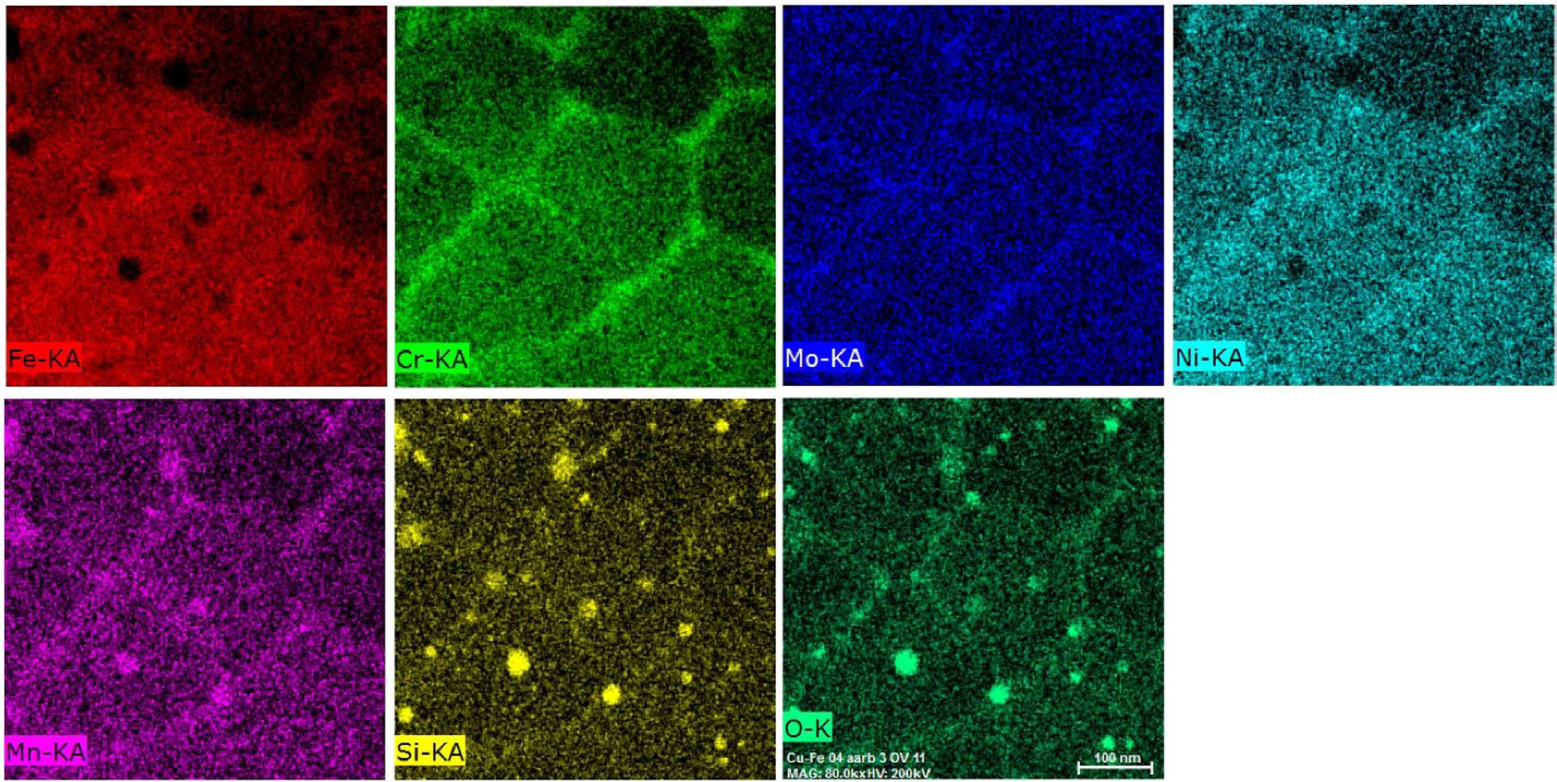
EDS-TEM chemical maps from an as-built 316L sample prepared using an EOS280 using factory-recommended build settings.

There is a further important aspect of the dislocation cells that are formed during thermal contraction of the SLM steels such as 316L during building. These cell boundaries interact with the solute distribution and can lead to microscale segregation behavior. This is shown in Figure 12, which is an energy dispersive spectroscopy (EDS) map made in TEM of the cell structures shown in Figure 10.

Clear segregation of Cr, Mo, Ni and Mn to the dislocation cell boundaries can be observed. This does not appear to be associated with deleterious Cr carbide formation, but nonetheless segregation is present, and the effect of this segregation of electrochemical properties must be investigated. The segregation is also likely to affect the dislocation-dislocation junction energy and strength and could lead to enhanced dislocation strengthening through a modification of α in the Taylor equation [53]. Indeed, the contribution of the as-built dislocation density to the overall flow stress may be even larger than that estimated above if the solute segregation shown in Figure 12 leads to increases in dislocation junction strengths. None of this has so far been investigated and requires work. This type of microscale segregation is something we will come back to in Section 3.2 in the context of maraging steels. There is a final comment to make about the TEM-EDS map of as-built 316L shown in Figure 12. A large number of Si and Mn-containing oxides may be observed. These same particles can also be seen in the bright-field TEM micrograph shown in Figure 10(b). These oxide particles form due to *in situ* oxidation during the SLM process due to residual oxygen in the build chamber. They are not present in any significant quantities in the starting powder for this material. They are 10–30 nm in size and highlight the very interesting possibilities that are available for using the build atmosphere both as an important processing variable and for *in situ* tailoring of the interstitial content of the steels. This possibility has recently been highlighted by Collins et al. [54] and Springer et al. [55], but if one carefully observes in TEM any SLM stainless steel containing Si, these oxides will be observed due to the high tendency for Si to oxidize. They have always been present in such steels prepared using SLM.

The stress/strain curves shown in Figure 11 for the SLM 316L compare very favorably with the annealed wrought or cast products. They are significantly stronger without obvious detrimental effects on the elongation. However, care must be taken because these steels prepared by SLM will contain some porosity, even if the processing conditions are controlled very well to keep it below 1%, and an unavoidable surface roughness that is inferior to most machined surfaces. Both of these characteristics will affect the damage properties such as fatigue.

**Figure 13.**
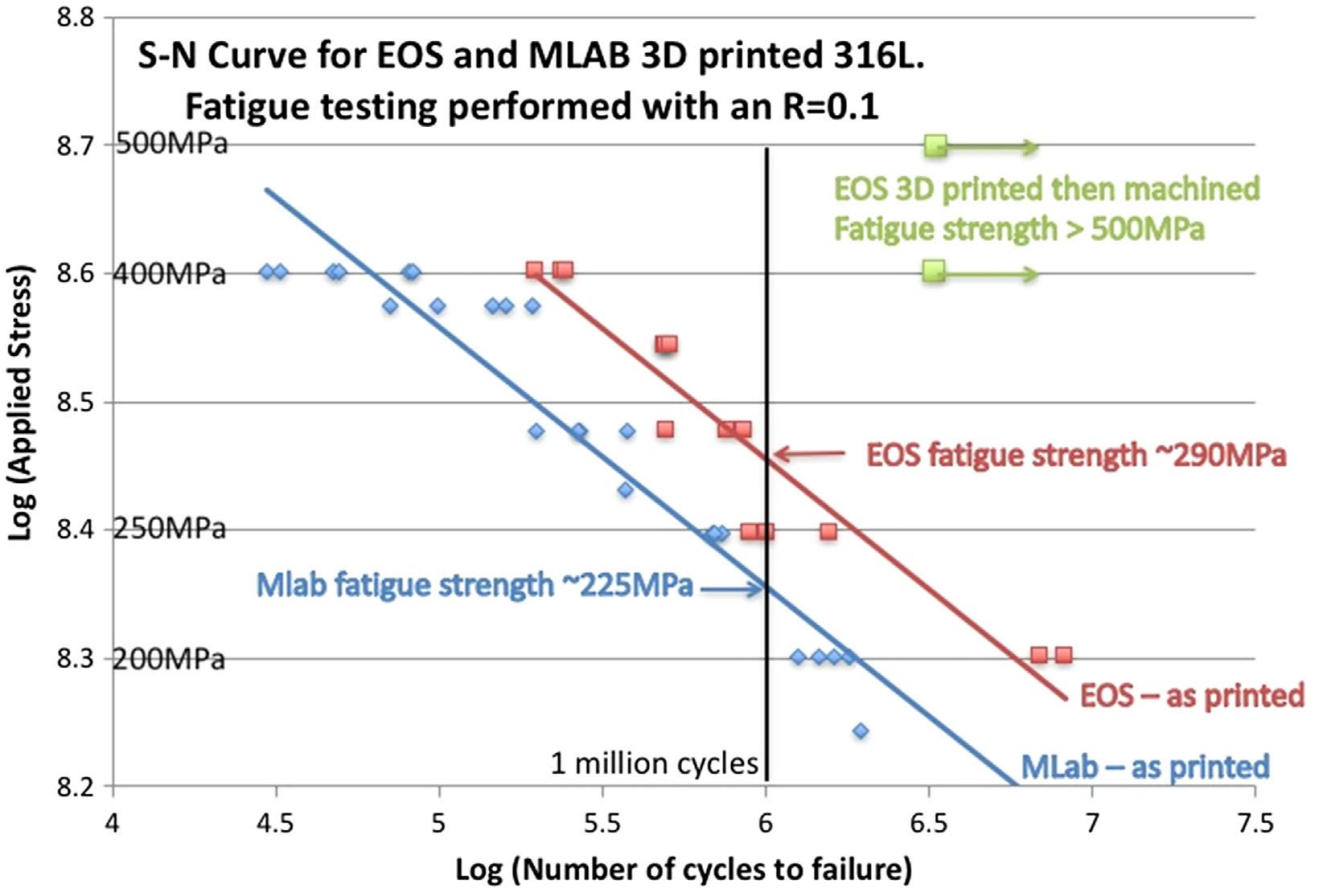
S-N curves for SLM prepares 316L. One batch of samples was prepared using a Concept Laser MLab and the other using an EOS M280. HCF sample geometry was directly printed without any post-printing treatments. The HCF tests were performed with R = 0.1.

Consider the high-cycle fatigue (HCF) S-N curves shown in Figure 13 for 316L tested under a R-ratio of 0.1 (tension-tension loading). The S-N curves for two batches of 316L prepared by SLM and tested in the as-built state are shown. One batch was printed on a Concept Laser MLab machine (Concept Laser, Lichtenfels, Germany) and the other on an EOS M280 (Electro-Optical Systems, Krailling, Germany). In both cases the final fatigue sample geometry was directly printed with no post-printing treatments applied. The two batches have very similar monotonic tensile responses, but their fatigue responses are significantly different – the EOS-printed samples are significantly better in fatigue and exhibit a fatigue strength at 10^6^ cycles of ~290 MPa, which compares favorably with the fatigue strength of wrought 316L under these cycling conditions of 250–290 MPa.

In this respect, one might be quite happy that the cyclic behavior of the SLM 316L is comparable to wrought 316L, but this must be qualified by the fact that the as-built SLM 316L is much stronger than the wrought product (~510 MPa vs ~290 MPa, Figure 11). For HCF performance, the fatigue strength usually scales with the tensile strength, and hence one might expect the SLM 316L to be much better in cyclic deformation than the wrought material. That it is not is because of at least two factors: one is the presence of residual porosity (as also highlighted in Section 2 for the fatigue of Ti-6Al-4V), and the other is because of the surface roughness inherent in the SLM process. For the EOS-printed 316L samples shown in Figure 13, the surface roughness is ~12–15 μm. We may test the relative effect of surface roughness and residual porosity by comparing the HCF performance of as-built HCF samples with those machined from as-built SLM cylinders prepared under the same conditions. Cylinders (with the same diameter as the heads of the HCF samples plotted in Figure 13) have been built on the EOS M280 at the same time as the HCF samples shown in Figure 13, and these cylinders were then machined into the same shape as the as-built HCF samples. These SLM 316L samples, containing a ‘machined’ surface finish, are also shown in Figure 13. For such samples, cycling under R=0.1 with a peak stress of 500 MPa is still not sufficient to break them after 5×10^6^ cycles (i.e. the fatigue strength is more than 500 MPa). This demonstrates the enhanced HCF performance that ‘could’ be obtained from the higher strength of SLM 316L compared to wrought 316L, if the surface finish in SLM could be improved. Whilst there is rightfully much focus on porosity because of its effects on damage, Figure 13 demonstrates that the fatigue performance is greatly compromised by the SLM surface finish and that efforts to improve this are also very important from the point of view of damage tolerance.

**Figure 14.**
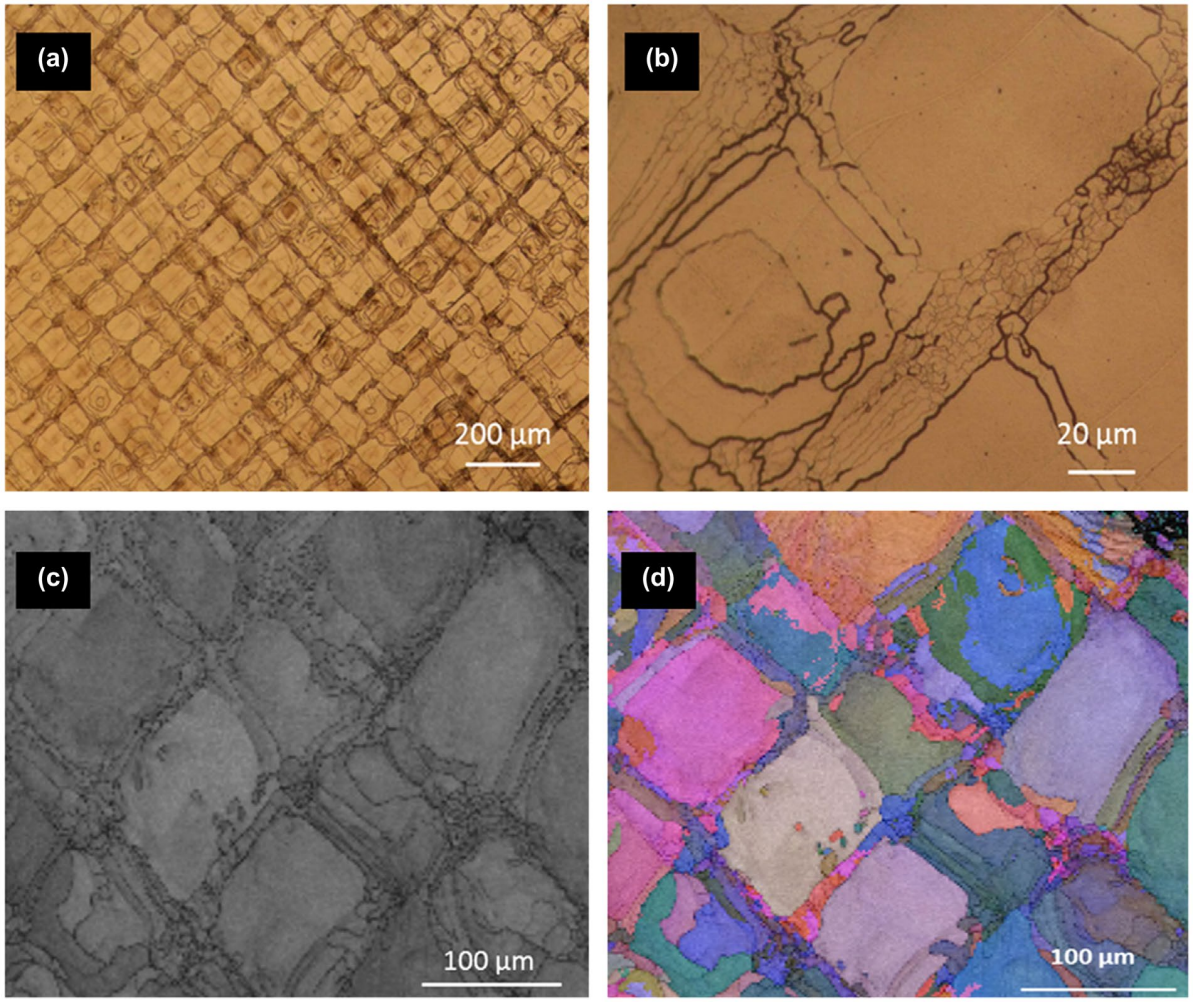
(a), (b) Optical microscopy images of SLM 25Cr super duplex steel taken at low (a) and high (b) magnification demonstrating the mosaic-type macrostructure. (b) Small grains around 1–5 μm within the mosaic boundary zones. (c) EBSD phase map showing existence of single-phase ferrite in the as-built state. (d) Grain orientation map of the same area showing existence of fine grains inside the tesserae (from Saeidi et al. [56]).

There is a second important aspect of the high dislocation densities observed in as-built steels. The dislocation densities observed correspond to large stored energies – the types of stored energies that typically drive recrystallization processes in metals. We may therefore expect that such structures should be relatively unstable and could be sensitive to the dissipation of heat through the already densified component during solidification of subsequent layers in SLM. This effect is actually already seen, although its origin has not been discussed. A series of optical and EBSD maps from a 25Cr super duplex stainless steel in the as-built SLM condition from Saeidi et al. [56] is shown in Figure 14. Although this composition is a duplex stainless steel, in the as-built state it is essentially 100% ferritic.

The images in Figure 14 are taken from a plane perpendicular to the build direction which was made on an EOS M270 machine. The interesting feature of these structures is the mosaic pattern observed. Large grains are surrounded by an array of small grains. The characteristic length scale of this pattern (100 μm) corresponds to the laser line spacing and is due to localized recrystallization of the as-built ferrite on subsurface layers due to the heat-affected zone from successive layers of building. This interesting feature of the microstructure is only possible because of the high dislocation density (and hence high stored energy) present in the as-built structure and highlights the possibilities of controlling grain structure by using the laser for *in situ* heat treatments. It will be possible to create controlled heterogeneous grain structures during building in this way, which can have interesting effects on the mechanical response (e.g. interesting effects on strain hardening from bimodal grain size distributions). This direction of creating architectured structures *in situ* remains almost unexplored and may also be used as a template for the nucleation of second phases during post-printing heat treatments. This will provide an opportunity to obtain multiphase steel architectures through SLM.

### 3.2. Phase stability in SLM steels

In Section 3.1, SLM of 316L was mostly discussed along with some consideration of 25Cr super duplex from the work of Saeidi et al. [54]. The as-built structures of these two steels are single phase – austenitic for 316L and ferritic for the duplex composition. Obviously, the duplex stainless steel requires a post-build heat treatment to obtain the duplex structure and therefore to obtain the combinations of strength and stress corrosion cracking resistance (SCC) which makes it useful.

However, other steels are multiphase in the as-printed SLM state. Examples are maraging steels. These steels are interesting in the context of additive manufacturing because the phases that appear are not necessarily those expected, the phase stability in the printed state appears to be different to that of their wrought counterparts and the reasons are not fully understood. In some cases, the stability may be altered because of microsegregation, similar to that shown in Figure 12, but, as we shall see, in other cases the phase stability is greatly modified and the origin is not understood.

Kempen et al. [57] and Jagle et al. [58] have both studied SLM of an 18Ni (300 grade) maraging steel. In the traditional wrought state these steels consist of a fine distribution of intermetallic precipitates formed by precipitation in a martensitic matrix. In principle, one might imagine these steels to be well disposed towards SLM because of the fast cooling rates and the need to form a starting martensitic microstructure. However, these authors demonstrated that the as-built state actually contains nearly 6% austenite and no precipitates, despite the repeated heating and cooling that might be expected to drive some precipitation during SLM. These authors also demonstrated that, during the post-build heat treatment to precipitate the strengthening intermetallics, the fraction of austenite grows from the retained austenite. The presence of the retained austenite is thought to be related to microscale segregation (like that shown in Figure 12) of austenite stabilizers during fabrication [59]. Jagle et al. [59] report that Kempen et al.’s [57] build was performed in a N_2_ atmosphere. Nitrogen is an austenite stabilizer and brings us to the important point of possible interstitial pick-up during additive manufacturing. In the same manner in which Ti alloys are sensitive to oxygen pick-up during additive manufacturing, steels are sensitive to nitrogen and carbon pick-up. Whilst this pick-up may be detrimental in some cases, one may also view the build atmosphere and a potentially controlled interstitial pick-up as an alloy design variable to be exploited. The formation of oxides (oxygen pick-up) is shown in Figures 10 and 12 in SLM 316L, but this may be extended to form different fractions of ferrite/martensite and austenite in SLM steels. By changing the atmosphere as a function of build time, architectured steels may be fabricated. The use of the build atmosphere as a design tool for SLM is only just commencing and provides great flexibility for the alloy designer. This possibility has been encouraged by Collins et al. [54] and Springer et al. [55].

A final example to demonstrate unexpected phase stability in SLM steels is from the work of Baek et al. [60]. These authors prepared samples of 304L using laser-aided direct metal tooling and compared their mechanical response with wrought 304L. As expected from the discussion on dislocation densities in Section 3.1, under monotonic testing the yield strength of the printed 304L was superior to that of the wrought product. However, these authors also mechanically tested their samples in a 10 MPa hydrogen atmosphere to compare the hydrogen embrittlement (HE) behavior of the additively manufactured and wrought 304L. The wrought 304L was embrittled by the hydrogen as is expected, but the AM 304L was largely unaffected. The HE resistance of the AM 304L is a surprising outcome, and the origins of this effect, and its generality, are not yet understood. However, a clue is found from examining the phase stability during deformation. Wrought 304L undergoes strain-induced martensite formation during tensile testing, and Baek et al. [60] demonstrate that this occurs in their experiments. However, the printed 304L did not transform during straining. The stability of the austenite in the additively manufactured 304L was significantly increased, and the origin is not known. It may well be that the large dislocation density that will be present in the as-built austenitic structure (Section 3.1) was sufficiently high to retard the martensite interface motion (but this is merely speculation). If so, it demonstrates another method (in addition to microsegregation and interstitial pick-up) of manipulating phase stability that may be exploited by alloy designers applying their trade to additively manufactured steels.

## 4. Microstructure and mechanical properties of high-entropy alloys processed by direct laser fabrication

In section 3.2, it was highlighted that phase stability in as-printed SLM multiphase steels differs from that of their wrought equivalents. This point is of prime interest for multi-principal element alloys (MPEAs) or high-entropy alloys (HEAs) which include a large range of compositional and microstructural complexity that is available for their development as structural materials [61]. HEAs are defined as alloys that have at least five principal elements in equimolar/equiatomic or near equimolar/equiatomic compositions and the concentration of each element is between 5% and 35%. The higher configurational entropy in HEAs stabilizes simple solid solutions, such as body-centered cubic (*bcc*), face-centered cubic (*fcc*), and hexagonal close-packed (*hcp*). However, in many cases the best balance of properties is achieved for microstructures consisting of a disordered solid solution phase and an ordered multicomponent precipitate phase. The configurational entropy does play a crucial role in deciding the final structure/phase, but often the competition with enthalpy dictates the final overall microstructure, hence sometimes these alloys are referred to as complex concentrated alloys (CCAs). HEAs with a single-phase solid solution can be a subcategory under CCAs, but for simplicity the term HEA will be used for all multi-principal element alloys discussed in this section.

Traditionally arc-melting [62–64] and spark plasma sintering (SPS) [65,66] have been the two main processing techniques employed to fabricate bulk HEAs. To successfully produce homogeneous bulk HEAs by arc-melting, extensive re-melting and intermittent ingot inversions are required, and powder alloying and refinement (typically via balling milling) is necessary when processing via the SPS route. In addition to these intensive processing requirements it may be argued that there are shape and size limitations of HEA components using these techniques. These are strong incentives toward the application of additive manufacturing to HEAs. AM of HEAs is beginning to receive attention (only two papers on SEBM [67] and SLM [68]), and a growing number of published studies report the microstructure and properties of HEAs processed by DLF. DLF is particularly attractive for the manufacture of HEAs because the need to pre-alloy metal powders can be avoided by the use of elemental powders as feed stock and their controlled supply from different hoppers to produce, *in situ*, chemically homogeneous alloys under optimized processing conditions.

The compatibility and advantage of AM of HEAs is also founded on the idea that laser-induced rapid solidification can avoid compositional segregation, restrict the formation of the brittle intermetallic compounds and lead to the strengthening effect by the grain refinement. In this section, we will discuss the phases, microstructures and mechanical properties of laser-printed and laser-cladded HEAs.

### 4.1. Bulk HEAs produced by SLM, SEBM and DLF

The bulk of the published literature shows LENS™ as being the most widely used additive manufacturing method for the production of HEAs. The biggest advantage LENS™ has over other systems is that it is a powder-*fed* system. This has the implication that gradation can be achieved simply by changing the powder flow rates from the different hoppers comprising the powder delivery system. Welk et al. [69], in 2013, first published the use of LENS™ for the additive manufacturing of a HEA, Al_1.5_CoCrCuFeNi. Their idea to use an additive manufacturing technique was based on the premise of avoiding slow cooling (common in cases using arc-melting or levitation melting) and hence to minimize the interdendritic segregation in the microstructure. Although the cooling rates are comparatively higher in direct laser deposition methods, the study did show the formation of dendritic grains. The presence of an ordered B2 structure and a disordered *bcc* matrix in these dendritic grains was confirmed via SEM, confocal TEM and scanning TEM studies. Kunce et al. then studied the use of additive manufacturing techniques to make HEAs for the purpose of hydrogen storage [70,71]. The premise for using additive manufacturing was the same as Welk et al. [69] to avoid slow cooling rates. The faster cooling rates obtained via direct laser metal deposition lead to a significant non-equilibrium solute-trapping effect, which in turn avoided the component segregation and relieved the solubility limitations. Two alloy systems of compositions, ZrTiVCrFeNi and TiZrNbMoV, were synthesized via the LENS™ system. Elemental powder blends were used. The compositions were chosen on their ability to form intermetallic phases which would be stable on exposure to annealing and hydrogen influence. While both alloys exhibited a dendritic two-phase structure, the ZrTiVCrFeNi alloy was made up of a dominant C14 Laves phase matrix with a minor amount of α-Ti solid solution, and the TiZrNbMoV showed dendrites of *bcc* solid solution surrounded by NbTi_4_-type phases. Both these alloys performed well for hydrogen storage. The use of LENS™-based additive manufacturing was also exploited by Choudhuri et al. [72] who used elemental powder blends to manufacture multi-component HEAs with ordered L2_1_ Heusler precipitates. The as-laser deposited sample was compared to an as-cast microstructure of the composition, AlCoCrCuFeNiTi, and revealed similar microstructures but at a much finer scale. This was attributed to the faster cooling rates obtained in the LENS™ process.

The most-studied high-entropy alloy system made via additive manufacturing has been based on the AlCoCrFeNi system. Kunce [73] used LENS™ and Fujieda [67] used SEBM. As the names suggest, while LENS™ is a laser-based process, SEBM is electron-based. The other major difference between these systems is that the LENS™ is a powder-*fed* process, compared to SEBM’s powder *bed* process. While Kunce concentrated more on the microstructural evolution (the alloy decomposed into dendrites and interdendrites; both composed of Fe–Cr-rich *bcc* precipitates in an Al–Ni-rich B2 matrix), Fujieda showed that SEBM-processed HEA had better mechanical properties than the same material in the as-cast state, with ductility and fracture strength both being high.

Brif et al. [68], Joseph et al. [74] and Sistla et al. [75] all used variants of the aforementioned compositions in their work involving additive manufacturing methods. SLM CoCrFeNi tensile test specimens were manufactured by Brif et al. [68] using gas-atomized powder. This gives rise to a fully dense microstructure which is 100% *fcc*. The yield strength of as-built SLM CoCrFeNi (600 MPa) has tripled compared with its cast equivalent (188 MPa), while the ductility remains high (32% elongation, and 50% for the as-cast), and the UTS reaches 745 MPa (457 MPa for the as-cast). This considerable enhancement of the strength is unlikely to be explained by the refinement of the microstructure.

**Figure 15.**
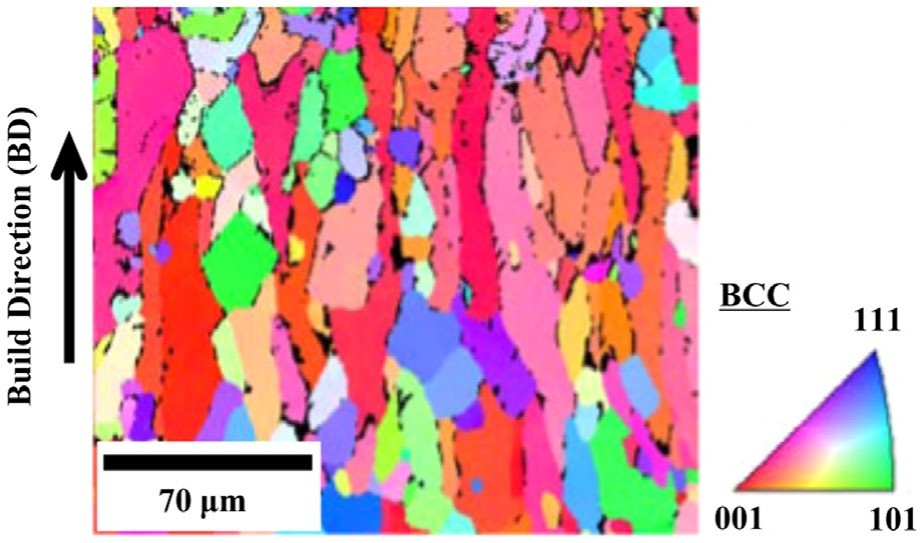
EBSD map (inverse pole figure) from as-built AlCoCrFeNi HEA prepared using SEBM Arcam A2X system (Arcam, Mölndal, Sweden) [67].

Fijieda et al. [67] used SEBM and gas-atomized powder to fabricate AlCoCrFeNi specimens for compression tests. This gives rise to a duplex *bcc* + *fcc* microstructure with elongated grain along the build direction (Figure 15). Anisotropy of the compressive properties has been reported. Segregation of Cr and Fe to the grain boundaries is observed. SEBM AlCoCrFeNi exhibits superior ductility (14%–26% elongation) and lower yield strength (1015 MPa) than the as-cast equivalent (5% and 1308 MPa, respectively). The enhanced plasticity is attributed to the grain refinement and the existence of the *fcc* phase in the SEBM specimen, which is not the case in the as-cast sample.

By varying the percentage of Al in the alloy Al_x_CoCrFeNi (x = 0.3, 0.6, 0.85), Joseph [74] achieved three completely different microstructures with the help of direct metal deposition. With an increasing amount of Al, the microstructure shifted from *fcc* to *fcc*/*bcc* to completely *bcc*. Mechanical properties of these additively manufactured samples, measured by compression testing, were comparable to the properties of as-cast alloys. While there was an increase in the overall strength with an increase in the Al percentage, the ductility decreased. While Joseph varied the composition of only Al, a change in both the Al and Ni compositions was considered out by Sistla et al. [75]. The alloys they studied, produced via direct laser sintering, were Al_x_FeCoCrNi_(2-x)_ (x = 0.3, 1). The transition of solid solution from a *bcc* to *fcc* structure via a change in the ratio of Al to Ni was the premise of this study. They reported that, with a decreasing Al content, the structure changed from a hard and brittle α+B2 structure to a hard and *relatively less brittle* B2+α+L1_2_ structure.

**Figure 16.**
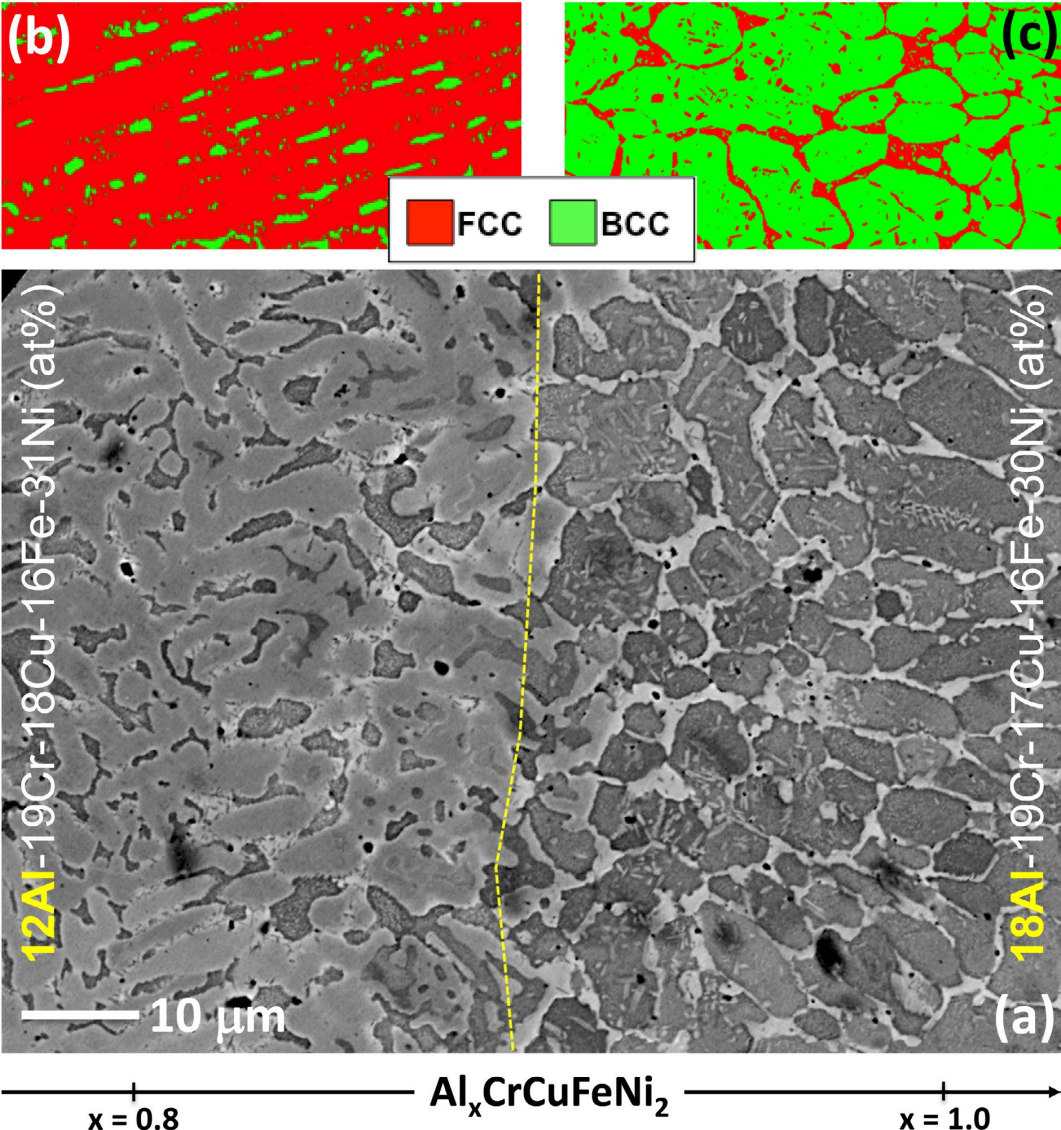
(a) Backscattered SEM image showing microstructural transition from x = 0.8 to x = 1.0 along compositionally graded LENS-deposited Al_x_CrCuFeNi_2_. EBSD phase maps showing fcc (red) and bcc (green) distribution in (b) x = 0.8 and (c) x = 1.0 [76].

A slightly modified alloy system was investigated by Borkar et al. [76], followed by Choudhuri et al. [77]. A single deposit of Al_x_CuCrFeNi_2_ (0 < x < 1.5) alloy was made via LENS™. The major difference in these studies, compared to earlier work, was that these investigations used a single deposit with compositional gradation along the build length. This approach permitted a detailed assessment of the transition in microstructure along the same alloy gradient, from a predominately *fcc* solid solution, to *fcc*/L1_2_ to mixed *fcc*/L1_2_ + *bcc*/B2 and finally to predominantly *bcc*/B2 as the Al content increases from 0 to 1.5 (molar fraction) (Figure 16). This change in microstructure and phase constitution was accompanied by a corresponding progressive increase in microhardness with increasing Al content, clearly indicating that the *bcc*/B2 microstructure is substantially harder than the *fcc*/L1_2_ microstructure. The corresponding change in magnetic properties with increasing Al content is also rather interesting, with the low Al-containing *fcc*/L1_2_ regions being weakly ferromagnetic, while the *bcc*/B2 regions with higher Al content are strongly ferromagnetic with high saturation magnetization (M_s_) but relatively soft with low coercivity (H_c_). Choudhuri et al. [77] in their work pinpointed the exact composition where the transformation occurs from *fcc* to *bcc* (~ 12 at.% Al), and the solidification sequence and subsequent solid-state transformations were also investigated and coupled with solution thermodynamic computations.

### 4.2. Laser-cladded HEA coatings

Laser cladding is the application of a thick coating (above 1 mm) which melts and bonds to the substrate [78–80]. The coating material can be fed as powders in a gas stream or as a wire, or preplaced as elemental or pre-alloyed powders, to be melted by the laser beam with a thin layer of the substrate. Laser cladding is used to tailor the surface properties of engineered materials (including steels, Ni-, Co-, Cu-, Al- and Ti-based alloys) exposed to severe environments. It provides wear and abrasion resistance, corrosion resistance and thermal resistance, while combining with the required bulk properties. Typical uses include coatings of steam turbine blades and turbine blades of aero-engines, hot rollers, hot gears, offshore drilling, cutting tools, valve components, etc. This process can also be used for maintenance and repair of damaged surfaces. The most commonly used coating materials are Co- and Ni-based superalloys and stainless steels [81–84]. Alloying elements added to tune the properties include Cr, V, Mo, Ti, Mn, W and C. Various ceramics powders (WC, TiC, SiC, Al_2_O_3_, Cr_2_O_3_, BN, TiN, etc.) can also be added to enhance the hardness and wear resistance but reduce the toughness (embrittlement). Recently, HEAs have been exploited as a material coating option by laser cladding. The studies published are more numerous than for bulk and thus provide complementary results and insights on the microstructure development in laser-melted HEAs, considering the similarities between laser cladding and DLF.

#### 4.2.1. Relationship between processing conditions and cladding quality of HEA coatings

Cladding process conditions affect the microstructure, macrostructure, surface morphology and overall quality of laser-cladded coatings. The main processing parameters are laser power (P), laser beam size (D) and laser scanning velocity (V) and combined parameters such as the effective energy per unit area E (J/mm^2^) and the powder deposition density PDD (g/mm^2^) [78]:



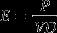





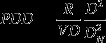



where R is the powder feed rate (g/min) and D_N_ is the nozzle diameter.

Table 1 summarizes the laser parameters applied for HEA coatings. Laser power, beam diameter, scanning velocity and effective energy applied for HEAs coatings range between 0.4–3 kW, 0.6–4.5 mm, 1.7–12 mm/s and 46–208 J/mm^2^. These conditions are typically used for successful clads of others metals and metal/ceramic composites coating [85].

**Table 1.** Summary of the processing conditions applied to laser cladding of HEA coatings.

Alloy composition	Preparation	Substrate	P (kW)	D (mm)	V (mm/s)	E (J/mm^2^)	t (mm)	Ref
AlB_0.5_CoCrCuFeMoNiSi	Pre-coating	Carbon steel	2	4.5	6.7	0.1	1.5	119
AlCoCrFeNi	Pre-coating	304 SS	2	3	3–7	0.1–0.2	0.5	95
AlCoCrFeNi	Pre-coating	1100 Al	0.7	0.6	1.7	0.7	0.5	117
Al_3_CoCrFeNi	Pre-coating	Q235 steel	2	4.5	5	0.1	1.2	107
Al_2_CoCrFeNiSi	Pre-coating	Q235 steel	2	4.5	6.7	0.1	1.2	108
AlCoCr(Cu)FeNiSi_0.5_	Pre-coating	AZ31 Mg	2	4	10	0.1	2	97
AlCoCrCu_0.9_FeNi	Pre-coating	AZ91D Mg	3	4	10	0.1	1	118
AlCoCrCuFe	Pre-coating	Q235 steel	3	4	3–6	0.1–0.3	0.8	105
(Al)CoCrCuFeNi	Pre-coating	AISI 1045 steel	1.4–1.8	3	8–12	0.05–0.1	1.4	87, 88
AlCoCrCuFeNi	Fed	Mg	0.3	1	2	0.2	0.3	96
Al_2_CoCrCuFeNi	Pre-coating	H13 steel	0.4	4	1–7	0.01–0.1	0.15	94
Al_2_CoCrCuFeNi(Ti)	Pre-coating	Q235 steel	2.5	4	3	0.2	1	93
Al_2_CoCrCuFe(Ni)Ti	Pre-coating	Q235 steel	2.5	4	3	0.2	0.8	104, 105
AlCoCrFeNiSiTi +C, B and Y_2_O_3_	Pre-coating	Q235 steel	1.38	5	6	0.05	2	100
AlCoCrFe_6_NiSiTi	Pre-coating	Q235 steel	2	4.5	6.7	0.1	1.7	101, 109
AlCoCrNiTiV	Pre-coating	Ti-6Al-4V	2	3	40	0.02	0.8	102
Al_2_CrFe(Mo)Ni	Pre-coating	SS	0.9	4	4	0.1	1.2	103
AlCrFeNiTa	Fed	305 SS	0.6	1.5	5	0.1	0.8	112
AlCrSiTiV	Fed	Ti-6Al-4V	2	2.5	3	0.3	2.5	86, 115
CoCrCuFeNi	Pre-coating	Q235 steel	2	4.5	6.7	0.1	1.2	106, 88
CoCrFeMnNi	Pre-coating	A36 steel	2	4	2	0.3	2	110
CrFeMoTiW	Pre-coating	Q235 steel	2	3	6	0.1	1.2	116

The most common defects in laser clads are unmelted powder particles, non-uniform and discontinuous clad tracks, and cracking. They are related to the process conditions. Unmelted powder particles occur when effective energy input is too low (i.e. high speed and low power) and/or when the powder feed rate is too high. The surface morphology and waviness of the clad layer depends on the overlap ratio, 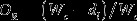 where d_*l*_ is the lateral displacement of successive tracks and W_*c*_ is the clad width (ideally equal to the laser spot size) which increases with the effective energy input. Good surface quality of the AlCrSiTiV and Al_x_CoCrCuFeNi coatings were prepared by Huang et al. [86] and Ye et al. [87] with overlap ratio ranging from 33% to 50% (optimum overlap distance close to half of the clad width). Zhang et al. [88] reported that minor additions of alloying elements can improve the quality of the CoCrCuFeNi coating.

Clad coatings can present an important network of cracks due to high residual stresses which arise from thermal mismatch and the rapid cooling process. The formation of cracks is enhanced when crack-sensitive coating materials are used, and when the substrate and the coating exhibit too dissimilar thermal expansion coefficients and Young’s moduli. As a rule, the coating materials should have a lower thermal expansion than the substrate to favor compressive stresses [89]. As shown by Huang et al. [86], cracking can also be avoided in AlCrSiTiV coating by preheating the Ti-6Al-4V substrate, causing the cooling rates to decrease.

**Figure 17.**
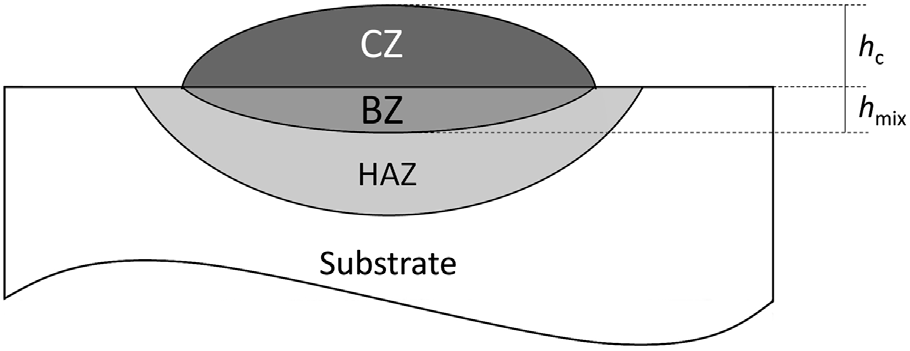
Clad dimensional characteristics and featured zones: cladding zone (CZ), bonding zone (BZ) and heat-affected zone (HAZ).



Figure 17 shows a schematic cross-section of a typical clad track. It consists of the cladding zone (CZ) with height h_c_, the bounding zone (BZ) with height h_mix_ and the heat-affected zone (HAZ). In the bounding zone, elements from the coating and the substrate material intermix with a certain ratio defined by the dilution rate. The dilution rate (d_*R*_) is an important factor affecting the properties of the cladding layer. It can be correlated to the clad geometry as shown in Figure 17 and calculated from:

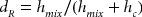



Dilution is necessary in order to create a strong metallurgical bond, but should be kept a low as possible (less than 5% [90]) to maintain the composition of the cladded coatings. As a general rule, an increase of the laser power and energy input contributes to the increase of the melting depth and dilution. Ye et al. [91] observed that high laser power (1800 W) and low scanning rate (4 mm/s) result in a large enrichment of the (Al)CoCrCuFeNi coating due to a dilution of Fe from the steel substrate. Even for lower laser power (600 W), Ocelik et al. [92] reported Fe enrichment of the AlCoCrFeNi and AlCrFeNiTa coatings by dilution (up to 70%) from the substrate. Other reports of laser cladding on steel substrates generally indicate a marginal increase of Fe [93,94] contents in Al_2_CoCrCuFeNi(Ti) and Al_2_CoCrCuFeNi coatings resulting from a slight dilution rate, despite different laser power applied (i.e. 2500 W and 400 W, respectively). Zhang et al. [95] pointed out a slight increase of Cr content in AlCoCrFeNi coating due to the mixing of Cr from the stainless steel substrate. Finally, the dilution between AlCoCrCuFeNi and AlCoCr(Cu)FeNiSi_0.5_ coatings and Mg substrate was investigated by Yue et al. [96,97]. It was found that the HEA clad and the Mg substrate were only slightly diluted by the Mg (7 at.%) and the Cu (2 at.%), respectively. The reason evocated for such limited dilution was the high viscosity and sluggish diffusion rates of the HEA melts. It can be noticed that all authors reported a depletion of the Al content in the cladded HEA coatings due to its evaporation from the melt pool.

The rapid cooling rates (10^4^–10^6^K/s) [98] induced by laser cladding and the temperature gradient between the bottom and the top of the melt pool lead to a typical microstructure of cladded coating consisting of columnar grains at the bounding zone and equiaxed grain at the cladding zone. This columnar-to-equiaxed transition of crystal growth found in laser cladding is controlled by the temperature gradient and the solidification rate in the same way discussed in Section 2 for Ti-6Al-4V (Figure 4). In the region close to the substrate, the high temperature gradient and low solidification rate cause a directional growth of columnar grains perpendicular to the interface. At the bottom of the melt pool, the solidification rate increases to a value close to the laser scanning velocity, while the temperature gradient decreases, which promoted the growth of equiaxed grains [99].

The typical microstructure in the cladding zone is composed of dendritic and interdendritic regions with slight component segregation [88,93,94,100–103]. For alloys containing both Cr and Cu elements, a tendency for segregation is observed with interdendritic regions enriched in Cu and depleted in Cr compared to the dendritic phase [88,94,104–106]. When alloys include Al, Cr, Fe and Ni, the solidified microstructure is generally composed of dendrites enriched in Al and Ni, while Cr and Fe are enriched in interdendritic regions [103,107,108]. Segregation of Ti and Mn in grain boundary regions is also observed in AlCoCrFe_6_NiSiTi [101,109] and CoCrFeMnNi [110]. In comparison with the solidified microstructure of the HEAs prepared by conventional casting or arc-melting techniques, the interdendritic region generally is much smaller and the segregation is much less significant due to the high cooling rate of the laser cladding method [106,109].

**Figure 18.**
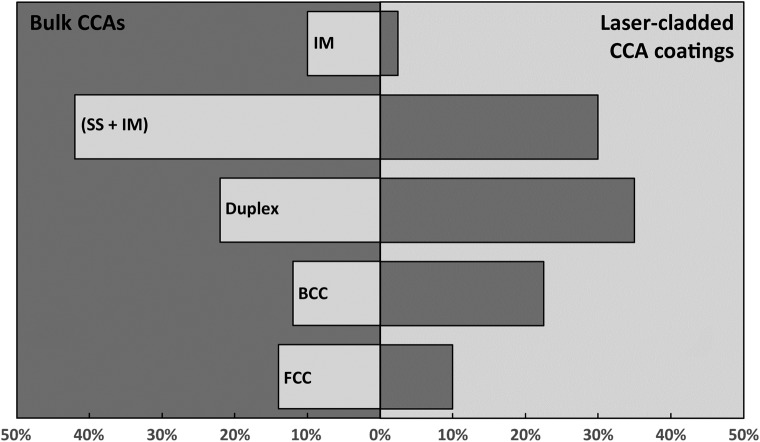
Microstructure classification by phase type. An IM has at least one IM phase, an (SS + IM) has at least one solid solutions and at least one IM, a Duplex has two solid solutions (bcc + fcc).

Table 2 and Figure 18 shows a microstructure classification by phase type for laser-cladded HEA coatings in comparison to bulk HEAs fabricated by conventional techniques. Almost all HEA coatings processed by laser cladding are from the 3d transition metal family, but one belonging to refractory metal has been reported. HEA clads and bulk HEAs share the same common elements. Disordered solid solutions (SS) and microstructures with a mixture of both disordered solid solution and intermetallic phases (SS+IM) represent 68% and 32% of reported microstructures, respectively. The most common phases are *bcc* (76%) and *fcc* (58%), followed by other IM. Common IM phases include B2. Single-phase *fcc* SS alloys are CoCrCuFeNi and CoCrFeMnNi, whereas single-phase SS *bcc* and duplex alloys contain *bcc* stabilizers such as Al, Ti, Si and *bcc* refractory metals. Half of the SS alloys are single-phase, with a higher percentage for disordered *bcc* (23% of the total) than *fcc* (10% of the total); the remaining (35% of the total) are duplex (*fcc* + *bcc*).

**Table 2.** Summary of properties and phases of laser-cladded HEA coatings.

Alloy composition	Density (kg/m^3^)	Hardness (HV)	Phases	Ref
AlB_0.5_CoCrCuFeMoNiSi	6713	1150	B2 + martensite	119
AlCoCrFeNi	6718		*bcc* + IM	117
AlCoCrFeNi	6718	510	*bcc*	95
Al_3_CoCrFeNi	5322	800	B2 + *fcc*	107
Al_2_CoCrFeNiSi	5156	900	*bcc* + B2	108
AlCoCr(Cu)FeNiSi_0.5_				97
AlCoCrCu0.5FeNiSi_0.5_	6324	800	*bcc*	
AlCoCrCuFeNiSi_0.5_	6507	700	*bcc* + *fcc*	
AlCoCrCu_0.9_FeNi	7041		*bcc*	118
AlCoCrCuFe	6753		*fcc* + *bcc*	105
(Al)CoCrCuFeNi				87
AlCoCrCuFeNi	7071	254	*fcc* + *bcc*	
Al_1.3_CoCrCuFeNi	6796	521	*fcc* + *bcc* + IM	
Al_1.5_CoCrCuFeNi	6631	398	*fcc* + *bcc* + IM	
Al_1.8_CoCrCuFeNi	6407	986	*fcc* + *bcc* + IM	
(Al)CoCrCuFeNi				91
AlCoCrCuFeNi	7071	390	*fcc* + *bcc*	
Al_1.3_CoCrCuFeNi	6796	540	*fcc* + *bcc*	
Al_1.5_CoCrCuFeNi	6631	640	*fcc* + *bcc*	
Al_1.8_CoCrCuFeNi	6407	660	*fcc* + *bcc*	
Al_2_CoCrCuFeNi	6271	687	*fcc* + *bcc*	
AlCoCrCuFeNi	7071			96
Al_2_CoCrCuFeNi	6271	400	*fcc*	94
Al_2_CoCrCuFeNi(Ti)				93
Al_2_CoCrCuFeNi	6271		*fcc* + *bcc*	
Al_2_CoCrCuFeNiTi_0.5_	6115		*fcc* + *bcc* + Laves phase	
Al_2_CoCrCuFeNiTi	5983		*fcc* + *bcc* + Laves phase	
Al_2_CoCrCuFeNiTi_1.5_	5872		*fcc* + *bcc* + Laves phase	
Al_2_CoCrCuFeNiTi_2_	5776		*bcc*	
Al_2_CoCrCuFe(Ni)Ti				104, 105
Al_2_CoCrCuFeTi	5655	900	*fcc* + *bcc*	
Al_2_CoCrCuFeNi_0.5_Ti	5828	920	*fcc* + *bcc*	
Al_2_CoCrCuFeNiTi	5983	960	*fcc* + *bcc*	
Al_2_CoCrCuFeNi_1.5_Ti	6124	1060	*fcc* + *bcc*	
Al_2_CoCrCuFeNi_2_Ti	6251	1100	*fcc* + *bcc*	
AlCoCrFeNiSiTi +C, B and Y_2_O_3_	5449	450	*fcc* + TiC + IM	100
AlCoCrFe_6_NiSiTi	6348	780	*bcc*	101, 109
AlCoCrNiTiV	5975		B2 + IM	102
Al_2_CrFe(Mo)Ni				103
Al_2_CrFeMo_0.5_Ni	5888	380	*bcc* #1 + *bcc#*2	
Al_2_CrFeMoNi	6292	445	*bcc*#1 + *bcc#*2	
Al_2_CrFeMo_1.5_Ni	6628	455	*bcc#*1 + *bcc#*2	
Al_2_CrFeMo_2_Ni	6911	678	*bcc#*1 + *bcc#*2	
AlCrFeNiTa	8967	860		112
AlCrSiTiV	4267	900	*bcc* + IM	86, 115
CoCrCuFeNi	8332	375	*fcc*	106, 88
CoCrFeMnNi	8028		*fcc*	110
CrFeMoTiW	9941	800	*bcc* + IM	116

This statistical comparison of the types of phases observed in HEAs processed by laser cladding and conventional solidification techniques [111], respectively, shows a prevalence of the *bcc* phase and duplex (*fcc* + *bcc*) microstructure over single *fcc*, and less IM phases. This over-representation of *bcc* may result from laser-induced rapid solidification as observed by Ocelik et al. [112], who compared the microstructure of (Al)CoCrFeNi prepared by conventional arc-melting with a laser-cladded coating technique and found that the formation of the *bcc* phase was favored over *fcc*. However, percentages of the reported microstructures are biased because 90% of the HEA coatings contain Al, and Al content is most of the time higher in clads than in bulk, which increases the number of single *bcc* phases (Al being a *bcc* stabilizer).

#### 4.2.2. Properties of laser-cladded HEA coatings

**Figure 19.**
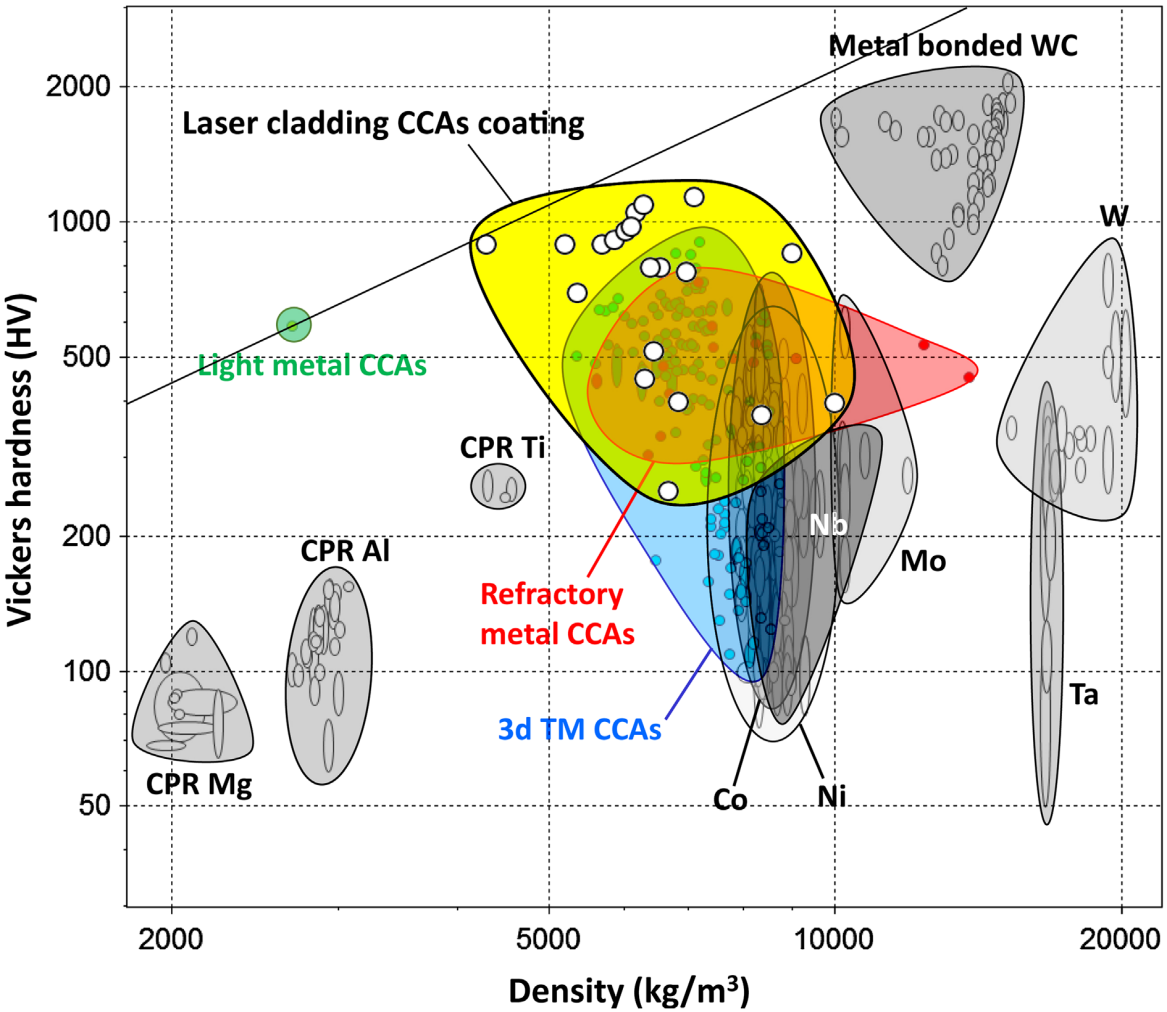
Materials property space for room temperature hardness vs density of laser cladding HEA coatings and conventional metal alloy coatings, ceramic particles reinforced (CPR) metallic matrix compatible with cladding or thermal spraying, and light-metal HEAs, 3d transition metal (TM) HEAs, and refractory metal HEAs. The dashed lines give performance index for uniaxial loading (HV/d). This chart was made using the CES EduPack, the Level 3 Aerospace database from Granta Design [114] and the HEA database published elsewhere [113].

**Figure 20.**
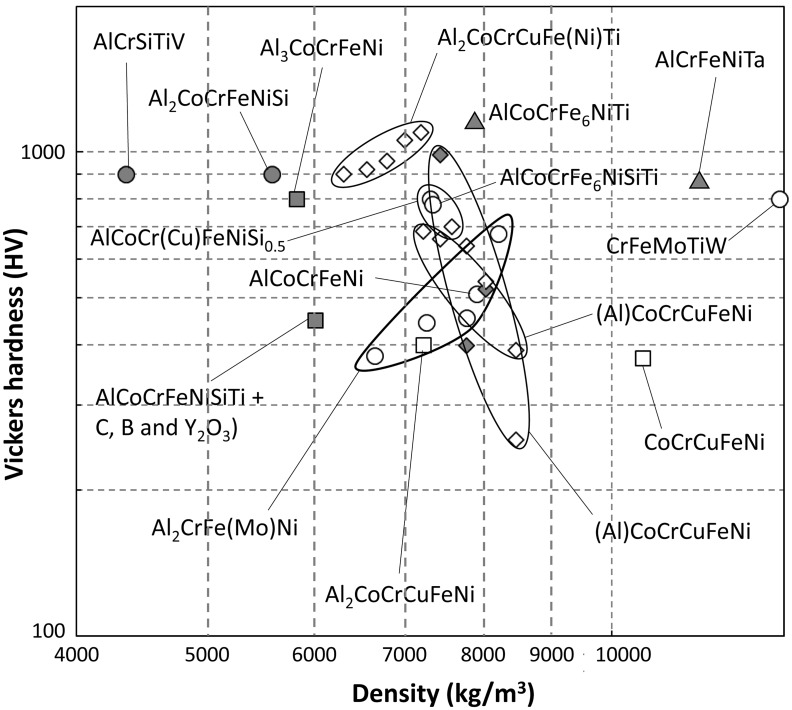
Detailed view of the materials property space for room temperature hardness vs density of laser cladding HEA coatings. Phases present are shown by squares for *fcc*, circles for *bcc*, diamonds for duplex (*fcc* + *bcc*) and triangles for other phases. SS and (SS + IM) are shown by open and closed symbols, respectively.

Figure 19 displays the materials property space where the room temperature hardness is plotted against density using logarithmic scales to compare HEAs coatings processed by laser cladding with alloys that are either currently used for surface coating (Co- and Ni-based alloys) or are compatible with cladding and thermal spraying (ceramic particle-reinforced Mg-, Al- and Ti-matrix, metal-bonded WC and refractory alloys). In addition, three families of bulk HEAs (light-metal HEAs, 3d transition metal HEAs and refractory metal HEAs) processed by conventional techniques (mainly arc-melting) are also shown on the same figure. This plot was made using a dedicated materials database for bulk HEAs [113], the CES EduPack Level 3 Aerospace database [114] and the published studies already cited, in addition to others [115–118] (see Table 2). Individual alloys (shown as open and closed circles) are enclosed in large bubbles that represent alloy families. The laser cladding HEA coatings are shown by white bubbles, 3d transition metal family of HEAs by blue-colored bubbles, refractory metal HEAs are shown by red bubbles and light-metal HEAs are shown by green bubbles; other commercial alloys and CRP metals are shown by gray bubbles. Figure 20 gives a more detailed view of HEA clads.

Laser-cladded HEAs coatings overlap with Co- and Ni-coatings and bulk 3d transition metal and refractory metal HEAs, and expend to higher hardness values filling empty areas of the materials landscape. The room temperature hardness of laser-cladded HEA coatings exceed the properties of all ceramic particle-reinforced Mg-, Al- and Ti-matrix, as well as Nb, Ta and Mo alloys. The best HEA clads outperform bulk HEAs which was attributed to the prevalence of the *bcc* phase, the grain refinement and the distribution of nano-sized precipitates resulting from the rapid cooling rate [87,91,93,107–109,112,119].

Laser-cladded HEAs coatings are marginally better than W alloys and do not compete with the best metal-bonded WC in hardness. However, the best HEAs coatings outperform W alloys and metal-bonded WC in applications where weight saving is a major requirement because they have considerably lower density than these refractory alloys and ceramic/metal composites. This feature is illustrated by the line representing the ratio of the hardness over the density in Figure 19. Materials above a performance index line have higher value of the specific hardness than those below, so harder and lighter coatings can be made from materials above the line.

Hard materials generally have low wear rates, which suggests the advantages of HEA coatings over conventional alloy and CRP coatings. Very few studies report wear volume or mass loss [86,103], wear rate [86,94], friction coefficient [86,87] and morphology of the worn surface [86,87,93,94,103] of HEA clads. Dry sliding wear tests at room temperature give friction coefficient and specific wear rate values of ~0.3 and ~2.10^−5^ mm^3^/Nm for AlCrSiTiV clad which is, respectively, 2 and 3 times lower than the Ti-6Al-4V substrate [86]. From EDS analysis of the worn surface of AlCrSiTiV clad, Huang et al. [86] attributed the enhanced abrasive and adhesive wear resistance to the combination of hard silicide secondary phase and ductile and tough *bcc* matrix which limit crack propagation. The abrasion resistance at room temperature of (Al)CoCrCuFeNi clad increases with Al content due to an increase of the hardness [87]. The wear rate at 500 °C for the Al_2_CoCrCuFeNi clad is 30% that of the H13 tool steel substrate [94]. The relative wear resistance at room temperature of Al_2_CoCrCuFeNi(Ti) coatings is about 3 times higher than for the Q235 steel substrate and almost independent of the Ti content despite the transition from a single-phase *fcc* for Ti_0_ to a single-phase *bcc* for Ti_2_ [93]. Room temperature sliding wear tests show that the mass loss of Al_2_CrFeMoNi coatings is only 50% of the stainless steel [103]. These papers show that laser-cladded HEA coatings significantly enhance the wear resistance of the substrate at room temperature and 500 °C. However, laser-cladded HEAs must be further characterized in order better to evaluate their potential as an attractive option for coating materials and to gain an understanding of the friction mechanism which provides an enhancement of the wear resistance in these alloys. Durability properties such as corrosion resistance in various environments, fracture toughness and response at elevated temperature are required for coating applications.

## 5. Conclusions

This review article has summarized and discussed the recent development of additive manufactured metals with a focus on the microstructures and mechanical properties of three different alloy families, i.e. Ti-6Al-4V, steels and high-entropy alloys.

In an as-printed SLM Ti-6Al-4V, even a small fraction of porosity (<1%) affects damage properties and a larger fraction (>5%) deteriorates the monotonic mechanical response. Whereas the interrelation between process parameters and consolidation of dense products has been identified, the influence of powder characteristics remains not sufficiently explored. Indeed, the powder size distribution and morphology are expected to influence the power absorption in a spatial manner. As a consequence, systematic efforts must be made to characterize more finely the powder and to incorporate these measurements into simulations.

From a macroscopic point of view, the columnar grain structure dominates, but a rigorous control of both the thermal gradient and the solidification rate velocity allows for developing fully columnar, fully equiaxed and mixed macrostructures. The preferential texture observed is mainly attributed to the preferential growth along the maximum thermal gradient that corresponds to the SLM built direction. It is obvious that a better knowledge about the local melt pool physics and the determination of the liquid/solid interfacial properties would lead to the definition of new strategies to design both texture and the macro-level microstructural features.

Due to high cooling rate, *β* phase transforms mainly to very fine α’-martensite upon cooling. The as-built samples by SLM are thus characterized by high strength, high yield strength but relatively low ductility. In that specific case, only some specific post-thermo-mechanical treatments may improve the balance between strength and ductility. For instance, both tensile strength and ductility can be strongly improved by the formation of a duplex α+*β* microstructure induced by a post-subcritical annealing. This shows a promising pathway to developing high-strength and ductile titanium alloys. It is also possible to conceive that such thermo-mechanical treatments can be carried out *in situ* during processing. Furthermore, it is of prime necessity to implement some tools to characterize the material in real time during processing. In the same way as for classical solidification, advanced technics such as synchrotron diffraction are expected to provide new insights into materials.

From a mechanical point of view, the tensile properties obtained approach and sometimes exceed properties obtained from a conventional process. However, these properties differ from one machine to another. This suggests that the link between process parameters and the resulting microstructure must be better understood. In the same vein, the presence of metallurgical defects, both the heterogeneity and anisotropy of microstructures, limit these processing techniques for fracture applications. However, the development of predictive models for mechanical and fracture properties opens up new opportunities for a widespread use of these advanced technics.

Examining stainless steels and maraging SLM steels, other important microstructural features and process considerations inherent to additive manufacturing were highlighted, including the large in-built dislocation density, the surface roughness, the phase stability and the build atmosphere.

In as-printed 316L stainless steel, the rapid solidification and anisotropic heat removal gives rise to a fully dense, 100% austenitic microstructure with fine (10 μm) and elongated grains. The most remarkable microstructural feature of the as-built microstructure is the high dislocation density (up to 10^15^ m/m^3^) equivalent to what would be obtained after severe plastic deformation. Quantitative rationalizations demonstrated that such a high in-built dislocation density can arise from thermal contraction strain during solidification and contribute primarily to the doubling of the yield strength for SLM 316L compared with the wrought 316L. Furthermore, solute microscale segregation at the dislocation cell boundaries is present, and its effect on dislocation junction strength deserves to be investigated. Another important and still unexplored aspect of the high dislocation densities observed in SLM 316L is that the large stored energy offers a means to design multiphase steel architectures by using the laser for *in situ* thermal treatments.

Damage properties of SLM steels was also discussed. The comparison of the cycle behavior of as-built HCF samples with those machined from as-built cylinders demonstrated that the surface roughness inherent to the SLM process compromises the fatigue performance and that efforts should be devoted to improving SLM surface finish.

A final comment was made about the phase stability in SLM steels. The phases in printed state appear to be different to those of their wrought equivalents. In an 18Ni maraging steel, microsegregation and interstitial pick-up from the build atmosphere affect the fractions of ferrite/martensite and austenite. This suggests an additional tunable parameter to manipulate the microstructure by changing the atmosphere during additive manufacturing.

Finally, an overview of the current state of additive manufactured high-entropy alloys was provided. HEAs represent a newly growing branch of the alloy family tree. Due to their compositional complexity and high concentration of alloying elements, the production of HEAs requires intensive processing using conventional techniques which limit the size and shape of HEA components. AM offers a cost-effective alternative technique in the context of HEAs, and published results demonstrated that HEAs are suitable with AM techniques. The large temperature gradient and rapid solidification lead to significant grain refinement and non-equilibrium solute-trapping effect, which in turn avoided the component segregation and relieved the solubility limitations, in printed HEAs compared with their cast counterparts.

DLF is particularly attractive because the need to pre-alloy metal powders can be avoided by the use of elemental powders as feed stock and their controlled supply from different hoppers, to produce, *in situ*, chemically homogeneous alloys under optimized processing conditions. A statistical comparison of the types of phases in HEAs produced by laser cladding compared with their cast counterparts showed a prevalence of the *bcc* phase and duplex microstructure. The hardness of 30 laser-cladded HEAs was compared with commercial alloys used for coating and bulk HEAs using materials property space. It was demonstrated that the best HEA clads outperform cast HEAs and commercial coating metals.

Besides the promising results from the viewpoint of the microstructure refinement and the enhancement of the mechanical properties of bulk and cladded coating HEAs, DLF (e.g. LENS^TM^) proved to be a powerful combinatorial tool to produce materials libraries with composition gradients along the build direction. This approach was successfully applied to various HEAs (e.g. Al_x_CuCrFeNi_2_) to assess the transition microstructure as the Al content increases along a single graded alloy. Such high-throughput experiments are particularly interesting for the development of HEAs which requires the exploration of the vast composition space in the central region of multidimensional phase diagrams.

SLM and SEBM of HEAs using pre-alloyed gas-atomized powder have received much less attention; however, they deserve further study as the tensile properties thus obtained appear better. For example, the yield strength of SLM CoCrFeNi HEA is 600 MPa compared with 274 MPa for the wrought (and 188 MPa for the as-cast) counterpart. This doubling of the yield strength is equivalent to what was observed in SLM 316L. Given the compositional similarity between 3d transition metal HEAs (such as CoCrFeNi) and stainless steels, the insights and future work highlighted in the present review for SLM 316L offer an interesting inspiration for the HEA field to move forward. Further focus should be devoted to evaluating the microstructure and tensile properties of additively manufactured HEAs with the aim of achieving an in-depth comprehension of the mechanisms at the origin of the microstructure development and the resulting mechanical properties.

## Disclosure statement

No potential conflict of interest was reported by the authors.

## Funding

This work on stainless steel was financed by Woodside Energy in the framework of the Woodside Innovation Centre located at Monash University, and by the Australian Research Council (ARC) in the form of a Linkage Project [LP160100918].

## References

[1] Gibson I, Rosen DW, Stucker B Additive manufacturing technologies: rapid prototyping to direct digital manufacturing, New York (NY): Springer; 2009.

[2] Zhang L-C, Attar H Selective Laser Melting of Titanium Alloys and Titanium Matrix Composites for Biomedical Applications: A Review. Advanced Engineering Materials. 2016;18:463–475.

[3] Louvis E, Fox P, Sutcliffe CJ Selective laser melting of aluminium components. Journal of Materials Processing Technology. 2011;211:275–284.

[4] Sun Z, Tan X, Tor SB, et al. Selective laser melting of stainless steel 316L with low porosity and high build rates. Materials and Design. 2016;104:197–204.

[5] Frazier WE Metal additive manufacturing: A review. Journal of Materials Engineering and Performance. 2014;23:1917–1928.

[6] Olakanmi EO, Cochrane RF, Dalgarno KW A review on selective laser sintering/melting of aluminium alloys powders: Processing, microstructure and properties. Progress in Materials Science. 2015;74:401–477.

[7] Herzog D, Seyda V, Wycisk E, et al. Additive manufacturing of metals. Acta Mater. 2016;117:371–392.

[8] Lewandowski JJ, Seifi M Metal additive manufacturing: a review of mechanical properties. Annu. Rev. Mater. Res. 2016;46:151–186.

[9] Available Materials for Metal Additive Manufacturing: Characteristics & Applications, Farinia Group, https://www.farinia.com/additive-manufacturing/3d-materials/characteristics-and-applications-of-available-metals-for-additive-manufacturing.

[10] Seifi M, Salem A, Beuth J, et al. Overview of Materials Qualification Needs for Metal Additive Manufacturing. JOM. 2016;68(3):747–764.

[11] Delgado J, Ciurana J, Sereno L Comparison of forming manufacturing processes and selective laser melting technology based on the mechanical properties of products. Virtual and Physical Prototyping. 2011;6:167–178.

[12] Yadroitsev P, Krakhmalev I Yadroitsava, Selective laser melting of Ti6Al4V alloy for biomedical applications: temperature monitoring and microstructural evolution. Journal of Alloys and Compounds. 2014;583:404–409.

[13] Sallica-Leva E, Jardini AL, Fogagnolo JB Microstructure and mechanical behavior of porous Ti–6Al–4V parts obtained by selective laser melting. Journal of Mechanical Behaviour of Biomedical Materials. 2013;26:98–108. 10.1016/j.jmbbm.2013.05.011 23773976

[14] Gong H, Rafi K, Gu H, et al. Analysis of defect generation in Ti-6V-4V parts made using powder bed fusion additive manufacturing processes. Additive Manufacturing. 2014;1–4:87–98.

[15] Das S Physical aspects of process control in selective laser sintering of metals. Advanced Engineering Materials. 2003;5:701–711.

[16] Attar H, Calin M, Zhang M, et al. Manufacture by selective laser melting and mechanical behaviour of commercially pure titanium. Materials Science and Engineering A. 2014;593:170–177.

[17] Gu D, Meiners W, Wissenbach K, et al. Laser additive manufacturing of metallic components materials, processes and mechanisms. International Materials Reviews. 2012;57:133–164.

[18] Zhang S, Wei Q, Cheng L, et al. Effects of scan line spacing on pore characteristics and mechanical properties of porous Ti6Al4V implants fabricated by selective laser melting. Materials and Design. 2014;63:185–193.

[19] Song B, Dong S, Zhang B, et al. Effects of processing parameters on microstructure and mechanical property of selective laser melted Ti6Al4V. Materials and Design. 2012;35:120–125.

[20] Gong H, Rafi K, Gu H, et al. Influence of defects on mechanical properties of Ti–6Al–4V components produced by selective laser melting and electron beam melting. Materials and Design. 2015;86:545–554.

[21] Aboulkhair NT, Maskery I, Ashcroft I, et al. The role of powder properties on the processability of Aluminium alloys in selective laser melting. Munich: Lasers in Manufacturing conference; 2015.

[22] Li R, Shi Y, Wang Z, et al. Densification behaviour of gas and water atomized 316L stainless steel powder during selective laser melting. Applied Surface Science. 2010;256:4350–4356.

[23] Yang S, Evans JRG Metering and dispensing of powder; the quest for new solid freeforming techniques. Powder Technology. 2007;178:56–72.

[24] Welsch G, Boyer R, Collings EW Materials Properties Handbook: Titanium Alloys. ASM International: Materials Park, OH; 1994 p. 517.

[25] Marshall GJ, Young WJ II, Thompson SM, et al. Understanding the Microstructure Formation of Ti-6Al-4V During Direct Laser Deposition via In-Situ Thermal Monitoring. Journal of the Minerals. 2016;68:778–790.

[26] Craig JE, Wakeman T., Grylls R, et al., Edition Hoboken, NJ: Wiley-TMS; 2011 p. 103–110.

[27] Kelly SM, Kampe SL Microstructural Evolution in Laser-Deposited Multilayer Ti-6Al-4V Builds: Part I. Microstructural Characterization, Metallurgical and Materials Transactions A. 2004;35A:1861–1867.

[28] Wang T, Zhu YY, Zhang SQ, et al. Grain morphology evolution behavior of titanium alloy components during laser melting deposition additive manufacturing. Journal of Alloys and Compounds. 2015;632:505–513.

[29] Kobryn PA, Semiatin SL Microstructure and texture evolution during solidification processing of Ti–6Al–4V. Journal of Materials Processing Technology. 2003;135:330–339.

[30] de Formanoir C, Michotte S, Rigo O, et al. Electron beam melted Ti–6Al–4V: Microstructure, texture and mechanical behavior of the as-built and heat-treated material. Materials Science and Engineering A. 2016;652:105–119.

[31] Sieniawski J, Ziaja W, Kubiak K, et al. Titanium Alloys—Advances in Properties Control, Chap In: Sieniawski J, Ziaja W, editors. Microstructure and Mechanical Properties of High Strength two-phase Titanium Alloys. Rijeka, Croatia: InTech; 2013.

[32] Ahmed T, Rack HJ Phase transformations during cooling in ➪+ 🢨 titanium alloys. Materials Science and Engineering A. 1998;243:206–211.

[33] Khalid Rafi H, Starr TL, Stucker BE A comparison of the tensile, fatigue, and fracture behavior of Ti–6Al–4V and 15-5 PH stainless steel parts made by selective laser melting. The International Journal of Advanced Manufacturing Technology. 2013;69:1299–1309.

[34] Vrancken B, Thijs L, Kruth J-P, et al. Heat treatment of Ti6Al4V produced by selective laser melting: microstructure and mechanical properties. Journal of Alloys and Compounds. 2012;541:177–185.

[35] Krakhmalev P, Fredriksson G, Yadroitsava I, et al. Deformation behavior and microstructure of Ti6Al4V manufactured by SLM. Physics Procedia. 2016;83:778–788.

[36] Simonelli M, Tse YY, Tuck C Further understanding of Ti-6Al-4V selective laser melting using texture analysis. Journal of Physics, Conference Series. 2012;371:1–4.

[37] Zaefferer S A study of active deformation systems in titanium alloys: dependence on alloy composition and correlation with deformation texture. Materials Science and Engineering A. 2003;344:20–30.

[38] Rafi K, Kharthik HNV, Gong H, et al. Microstructures and mechanical properties of Ti6Al4V parts fabricated by selective laser melting and electron beam melting. Journal of Materials Engineering and Performance. 2013;22:3873–3883.

[39] Qian M, Xu W, Brandt M, et al. Additive manufacturing and post processing of Ti-6Al-4V for superior mechanical properties. MRS Bulletin. 2016;41:775–782.

[40] Xu W, Brandt M, Sun S, et al. Additive manufacturing of strong and ductile Ti-6Al-4V by selective laser melting via in situ martensite decomposition. Acta Materialia. 2015: 74–84.

[41] Nalla RK, Boyce BL, Campbell JP, et al. Influence of microstructure on high-cycle fatigue of Ti-6Al-4V: Bimodal vs. lamellar structures. Metallurgical and Materials Transactions A. 2002;33A:899–918.

[42] Lü jering G, Albrecht J, Gysler A Titanium: Science and Technology In: Froes FH, Caplan I, editors. Mechanical properties of titanium alloys. Warrendale, PA: TMS; 1993p. 1635–1646.

[43] Van Hooreweder B, Moens D, Boonen R, et al Analysis of Fracture Toughness and Crack Propagation of Ti6Al4V Produced by Selective Laser Melting. Advanced Engineering Materials. 2012;14:92–97.

[44] Zhao X, Zhao X, Li S, et al. Comparison of the microstructures and mechanical properties of Ti–6Al–4V fabricated by selective laser melting and electron beam melting. Materials and Design. 2016;95:21–31.

[45] Edwards P, Ramulu M, Edwards P, et al. Fatigue performance evaluation of selective laser melted Ti–6Al–4V. Materials Science and Engineering A. 2014;598:327–337.

[46] Simonelli M, Tse YY, Tuck C Fracture mechanisms in high-cycle fatigue of selective laser melted Ti–6Al–4V. Key Engineering Materials. 2015;627:125–128.

[47] Leuders S, Thöne M, Riemer A, et al On the mechanical behaviour of titanium alloy TiAl6V4 manufactured by selective laser melting: fatigue resistance and crack growth performance. International Journal of Fatigue. 2013;48:300–307.

[48] Bian L, Thomson SM, Shamsaei N Mechanical Properties and Microstructural Features of Direct Laser-Deposited Ti-6Al-4V. Journal of Metals. 2015;629–638.

[49] Li P, Warner DH, Fatemi A, et al. Critical Assessment of the fatigue performance of additively manufactured Ti-6Al-4V and prospective for future research. International Journal of Fatigue. 2015;85:130–143.

[50] Qiu C, Adkins NJE, Attallah MM Microstructure and tensile properties of selectively laser-melted and of HIPed laser-melted Ti-6Al-4V. Materials Science and Engineering A. 2013;578:230–239.

[51] Rafi HK, Starr TL, Stucker BE A comparison of the tensile, fatigue, and fracture behaviour of Ti-6Al-4V and 15-5 PH stainless steel parts made by selective laser melting. The International Journal of Advanced Manufacturing Technology. 2013;69:1299–1309.

[52] Vilaro T, Colin C, Bartout JD As-fabricated and heat treated microstructures of the Ti–6Al–4V alloy processed by selective laser melting. Metallurgical and Materials Transactions A. 2011;42(2011):3190–3199.

[53] da Costa Teixeira J, Brechet Y, Estrin Y, et al., The strain hardening behaviour of supersaturated Al-Cu alloys, Proceedings of the 12th International Conference on Aluminium Alloys, Sept. 5-9th, 2010, Yokohama, Japan.

[54] Collins PC, Brice DA, Samimi P, et al. Microstructure control of additively manufactured metallic materials. Ann Rev Mater Res. 2016;46:63–91.

[55] Springer H, Baron C, Szczepaniak A, et al. Efficient additive manufacturing production of oxide- and nitride-dispersion-strengthened materials through atmospheric reactions in liquid metal deposition”. Materials and Design. 2016;111:60–69.

[56] Saeidi K, Kevetkova L, Lofaj F, et al. Novel ferritic stainless steel formed by laser melting from duplex stainless steel powder with advanced mechanical properties and high ductility. Materials Science and Engineering A. 2016;665:59–65.

[57] Kempen K, Yasa E, Thijs L, et al. Microstructure and mechanical properties of selective laser melted 18Ni-300 steel. Physics Proceedia. 2011;12:255–263.

[58] Jagle EA, Choi PP, van Humbeeck J, et al. Precipitation and austenite reversion behaviour of a maraging steel produced by selective laser melting. J. Mater Res. 2014;29:2072–2079.

[59] Jagle EA, Sheng Z, Kurnsteiner P, et al., Comparison of maraging steel micro- and nanostructure produced conventionally and my laser additive manufacture. Materials. 2017; 10 article 8. 10.3390/ma10010008 PMC5344583 28772369

[60] Baek SW, Song EJ, Kim JH, et al. Hydrogen embrittlement of 3-D printing manufactured austenitic stainless steel part for hydrogen service. Scripta materialia. 2017;130:87–90.

[61] Miracle D, Miller J, Senkov O, et al., Exploration and Development of High Entropy Alloys for Structural Applications. Entropy. 2014: 494–525.

[62] Yeh J, Chen S., Lin S., et al., Nanostructured High-Entropy Alloys with Multiple Principal Elements: Novel Alloy Design Concepts and Outcomes. Advanced Engineering Materials. 2004: 299–303.

[63] Tsai K, Tsai M, Yeh J Sluggish diffusion in Co–Cr–Fe–Mn–Ni high-entropy alloys. Acta Materialia. 2013: 4887-4897.

[64] Otto F, Yang Y, Bei H, et al. Relative effects of enthalpy and entropy on the phase stability of equiatomic high-entropy alloys. Acta Materialia. 2013: 2628-2638.

[65] Fang S, Chen W, Fu Z Microstructure and mechanical properties of twinned Al_0.5_CrFeNiCo_0.3_C_0.2_ high entropy alloy processed by mechanical alloying and spark plasma sintering. Materials and Design. 2014: 973-979.

[66] Sriharitha R, Murty B, Kottada R Alloying, thermal stability and strengthening in spark plasma sintered Al_x_CoCrCuFeNi high entropy alloys. Journal of Alloys and Compounds. 2014: 419–426.

[67] Fujieda T, Shiratori H., Kuwabara K, et al. First demonstration of promising selective electron beam melting method for utilizing high-entropy alloys as engineering materials. Materials Letters. 2015: 12–15.

[68] Brif Y, Thomas M, Todd I “The use of high-entropy alloys in additive manufacturing. Scripta Materialia. 2015: 93–96.

[69] Welk B, Williams R, Viswanathan G, et al. Nature of the interfaces between the constituent phases in the high entropy alloy CoCrCuFeNiAl. Ultramicroscopy. 2013: 193–199. 10.1016/j.ultramic.2013.06.006 23870861

[70] Kunce, Polanski M, Bystrzycki J Structure and hydrogen storage properties of a high entropy ZrTiVCrFeNi alloy synthesized using Laser Engineered Net Shaping (LENS). International Journal of Hydrogen Energy. 2013: 12180–12189.

[71] Kunce, Polanski M, Bystrzycki J Microstructure and hydrogen storage properties of a TiZrNbMoV high entropy alloy synthesized using Laser Engineered Net Shaping (LENS). International Journal of Hydrogen Energy. 2014: 9904–9910.

[72] Choudhuri D, Alam T, Borkar T, et al. Formation of a Huesler-like L21 phase in a CoCrCuFeNiAlTi high-entropy alloy. Scripta Materialia. 2015: 36–39.

[73] Kunce, Polanski M, Karczewskia K, et al. Microstructural characterisation of high-entropy alloy AlCoCrFeNi fabricated by laser engineered net shaping. Journal of Alloys and Compounds. 2015: 751–758.

[74] Joseph J, Jarvis T, Wu X, et al. Comparative study of the microstructures and mechanical properties of direct laser fabricated and arc-melted AlxCoCrFeNi high entropy alloys. Materials Science and Engineering: A. 2015: 184–193.

[75] Sistla H, Newkirk J, Liou F Effect of Al/Ni ratio, heat treatment on phase transformations and microstructure of Al_x_FeCoCrNi_2−x_ (x = 0.3, 1) high entropy alloys. Materials and Design. 2015;81:113–121.

[76] Borkar T, Gwalani B, Choudhuri D, et al. A combinatorial assessment of Al_x_CrCuFeNi_2_ (0 < x < 1.5) complex concentrated alloys: Microstructure, microhardness, and magnetic properties. Acta Materialia. 2016;116:63–76.

[77] Choudhuri D, Gwalani B, Gorsse S, et al. Change in the primary solidification phase from fcc to bcc-based B2 in high entropy or complex concentrated alloys. Scripta Materialia. 2017;127:186–190.

[78] Toyserkani E, Khajepour A, Corbin S Laser Cladding. CRC Press, 2004, 280 p., ISBN 9780849321726.

[79] Advanced surface coatings: a handbook of surface engineering, Rickerby D.S., Matthews A., Blackie, Published by Chapamm and hall, 1991.

[80] Pawlowski L. Thick laser coatings: A review. Journal of Thermal Spray Technology. 8; 1999: 279–295.

[81] Kathuria YP Some aspects of laser surface cladding in the turbine industry. Surface and Coatings Technology. 2000;132:262–269.

[82] Lusquinos F, Comesan R, Riveiro A, et al. Fibre laser micro-cladding of Co-based alloys on stainless steel. Surf. Coat. Technol. 2009;203:1933.

[83] Xuan HF, Wang QY, Bai SL, et al. A study on microstructure and flame erosion mechanism of a graded Ni–Cr–B–Si coating prepared by laser cladding. Surf. Coat. Technol. 2014;244(203):203.

[84] Cui YH, Guo ZX, Liu YH, et al. Characteristics of cobalt-based alloy coating on tool steel prepared by powder feeding laser cladding. Opt. Laser Technol. 2007;39:1544.

[85] Emamian Ali, Corbin Stephen F, Khajepour Amir Effect of laser cladding process parameters on clad quality and in-situ formed microstructure of Fe–TiC composite coatings. Surface & Coatings Technology. 2010;205:2007–2015.

[86] Huang C, Zhang Y, Vilar R, et al. Dry sliding wear behavior of laser clad TiVCrAlSi high entropy alloy coatings on Ti–6Al–4V substrate. Materials and Design. 2012;41:338–343.

[87] Ye X, Ma M, Cao Y, et al. The property research on high entropy alloy AlxFeCoNiCuCr coating by laser cladding. Phys. Procedia. 2011;12:303–312.

[88] Zhang H, Pan Y, He YZ Synthesis and characterization of FeCoNiCrCu high-entropy alloy coating by laser cladding. Mater. Design. 2011;32:1910.

[89] Dekumbis R Controlling Residual Stresses in Laser Cladded Coatings, Proceedings of the 6th International Conference Lasers in Manufacturing, Birmingham IFS; 1989.

[90] Takeda T, Steen WM, and West DRF In-situ Clad Alloy Formation. In: Cladding Laser Proceedings of the LIM 2. Birmingham: IFS Publications; 1985.

[91] Ye X, Ma M, Liu W, et al. Synthesis and Characterization of High-Entropy Alloy Al_X_FeCoNiCuCr by Laser Cladding. Advances in Materials Science and Engineering. 2011;485942:1–7.

[92] Ocelik V, Janssen N, Smith SN, and De Hosson JTHM Additive Manufacturing of High-Entropy Alloys by Laser Processing. JOM. 2016;68:1810–1818.

[93] Qiu XW, Zhang YP, Liu CG Effect of Ti content on structure and properties of Al_2_CrFeNiCoCuTi_x_ high entropy alloy coatings. J. Alloys and Compds. 2014;585:282–286.

[94] Liu XT, Lei WB, Li J, et al. Laser cladding of high-entropy alloy on H13 steel. Rare Met. 2014;33(6):727–730.

[95] Zhang S, Wu CL, Zhang CH, et al. Laser surface alloying of FeCoCrAlNi high entropy alloy on 304 stainless steel to enhance corrosion and cavitation erosion resistance. Optics & Laser Technology. 2016;84:23–31.

[96] Yue TM, Xie H, Lin X, et al. Solidification behavior in laser cladding of AlCoCrCuFeNi high entropy alloy on magnesium substrate. J Alloys Compd. 2014;587:588–593.

[97] Yue TM, Zhang H Laser cladding of FeCoNiCrAlCu_x_Si_0.5_ high entropy alloys on AZ31 Mg alloy substrates. Materials Research. 2014;18:624–628.

[98] Zhang H, Pan Y, He Y-Z, et al. Application Prospects and Microstructural Features in Laser-Induced Rapidly Solidified High-Entropy Alloys. JOM. 2014;66:2057.

[99] Yue Tai M., Xie Hui, Lin Xin, et al., Microstructure of Laser Re-Melted AlCoCrCuFeNi High Entropy Alloy Coatings Produced by Plasma Spraying, Entropy 15 (2013) 2833–2845.

[100] Guo Y, Che S, Yuan Z, et al. FeCoNiAlTiCrSi high entropy alloy coating prepared by laser cladding, 5th international conference on Information Engineering for Mechanics and Materials (ICIMM. Huhhot. 2015;2015:600–604.

[101] Zhang H, Pan Y, He Y Effects of annealing on the microstructure and properties of 6FeNiCoCrAlTiSi high-entropy alloy coating prepared by laser cladding. Journal of Thermal Spray Technology. 2011;20:1049.

[102] Cai Z, Jin G, Cui X, et al. Synthesis and microstructure characterization of Ni-Cr-Co-Ti-V-Al high entropy alloy coating on Ti-6Al-4V substrate by laser surface alloying. Mater Charact. 2016;120:229–233.

[103] Wu W, Jiang L, Jiang H, et al. Phase evolution and properties of Al_2_CrFeNiMo_x_ high entropy alloys coatings by laser cladding. J Thermal Spray Tech. 2015;24(7):1333–1340.

[104] Qiu X-W, Zhang YP, He L, et al. Microstructure and corrosion resistance of AlCrFeCuCo high entropy alloy. J Alloys Compd. 2013;549:195–199.

[105] Qiu XW, Liu CG Microstructure and properties of Al_2_CrFeCoCuTiNi_x_ high entropy alloys prepared by laser cladding. J Alloys Compd. 2013;553:216–220.

[106] Zhang H, He YZ, Pan Y, et al. Thermally stable laser cladded CoCrCuFeNi high-entropy alloy coating with low stacking fault energy. J Alloys Compd. 2014;600:210–214.

[107] Zhang H, He YZ, Pan Y, et al. Synthesis and characterization of NiCoFeCrAl3 high entropy alloy coating by laser cladding. Adv Mat Res. 2010;97–101:1408.

[108] Zhang H, He YZ, Pan Y, et al. Phase selection, microstructure and properties of laser rapidly solidified FeCoNiCrAl2Si coating. Intermetallics. 2011;10:1130.

[109] Zhang H, Pan Y, He Y, et al. Microstructure and properties of 6FeNiCoSi-CrAlTi high-entropy alloy coating prepared by laser cladding. Appl Surf Sci. 2011;257:2259–2263.

[110] Ye Q, Feng K, Li Z, et al. Microstructure and corrosion properties of CrMnFeCoNi high entropy alloy coating. Applied Surface Science. 2017;396:1420–1426.

[111] Miracle DB, Senkov ON A critical review of high entropy alloys and related concepts. Acta Mater. 2017;122:448–511.

[112] Ocelik V, Janssen N, Smith SN, et al. Additive manufacturing of high entropy alloys by laser processing. JOM. 2016;68(7):1810–1818.

[113] Gorsse S, Miracle D, Senkov O Mapping the world of complex concentrated alloys. Acta mater. 2017;135:177–187.

[114] CES EduPack 2016, Granta Design Ltd. Cambridge UK (www.grantadesign.com/education).

[115] Huang C, Zhang Y, Shen J, et al. Thermal stability and oxidation resistance of laser clad TiVCrAlSi high entropy alloy coatings on Ti-6Al-4V alloy. Surf. Coat. Technol. 2011;206:1389.

[116] Zheng B, Liu QB, Zhang LY Microstructure and properties of MoFeCrTiW high-entropy alloy coating prepared by laser cladding. Advanced Materials Research. 2013;820:63–66.

[117] Katakam S, Joshi SS, Mridha S, et al. Laser assisted high entropy alloy coating on aluminum: microstructural evolution. J Appl Phys. 2014;116:104906.

[118] Huang KJ, Lin X, Wang YY, et al. Microstructure and corrosion resistance of Cu_0.9_NiAlCoCrFe high entropy alloy coating on AZ91D magnesium alloys by laser cladding. Materials Research. 2014;18:1008.

[119] Zhang H, He Y, Pan Y Enhanced hardness and fracture toughness of the laser-solidified FeCoNiCrCuTiMoAlSiB_0.5_ high-entropy alloy by martensite Strengthening. Scr Mater. 2013;69:342–345.

